# Randomised trial of delivering co-located, personalised stop-smoking support within lung cancer screening: the YESS study

**DOI:** 10.1136/thorax-2025-224217

**Published:** 2026-03-13

**Authors:** Rachael L Murray, David Baldwin, Kate Brain, John Britton, Christos V Chalitsios, Philip A Crosbie, Sarah Lewis, Grace McCutchan, Parrott Steve, Samantha Quaife, Rebecca Thorley, Qi Wu, Alexandra Ashurst, Rebecca J Beeken, Harriet Copeland, Rhian Gabe, Neil Hancock, Catriona S Marshall, Richard D Neal, Lucy R Porter, Suzanne Rogerson, Harriet Dorothy Quinn-Scoggins, Irene Simmonds, Andy Sloss, Matthew E J Callister

**Affiliations:** 1 School of Medicine, University of Nottingham, Nottingham, UK; 2 Nottingham University Hospitals NHS Trust—City Campus, Nottingham, UK; 3 Division of Population Medicine, Cardiff University, Cardiff, UK; 4 Division of Cancer Sciences, The University of Manchester, Manchester, UK; 5 Wales Cancer Research Centre, Cardiff University, Cardiff, UK; 6 Department of Health Sciences, University of York, York, UK; 7 Centre for Cancer Prevention, Wolfson Institute of Preventive Medicine, Barts and The London School of Medicine and Dentistry, Queen Mary University of London, London, UK; 8 Department of Radiology, Leeds General Infirmary, Leeds, UK; 9 Leeds Institute of Health Sciences, University of Leeds, Leeds, England, UK; 10 Centre for Cancer Prevention, Wolfson Institute of Preventive Medicine, Queen Mary University of London, London, UK; 11 University of Exeter, Exeter, UK; 12 Department of Research and Innovation, Leeds Teaching Hospitals NHS Trust, Leeds, UK; 13 PRIME Centre Wales, Cardiff University, Cardiff, UK; 14 Patient Representative, Leeds, UK; 15 Department of Respiratory Medicine, Leeds Teaching Hospitals NHS Trust, Leeds, UK

**Keywords:** Smoking cessation, Lung Cancer

## Abstract

**Introduction:**

Lung cancer screening (LCS) with low-dose CT offers a teachable moment for smoking cessation (SC), but the optimal way to implement SC within LCS is unclear. The Yorkshire Enhanced Stop Smoking (YESS) study assessed the efficacy of a personalised stop-smoking intervention delivered alongside LCS.

**Methods:**

Opt-out, co-located SC support, comprising nicotine replacement therapy/e-cigarettes/pharmacotherapy and behavioural support, was offered to all individuals who currently smoked attending for LCS. Four weeks later, participants were offered recruitment to a randomised controlled trial of continued standard best practice (SBP) versus a personalised SC support package, including a booklet containing CT images of participants’ own heart and lungs, annotated where appropriate to highlight emphysema or coronary artery calcification and scripted communication delivered by a smoking cessation practitioner.

**Results:**

1003 people were recruited; 52.5% were allocated to the intervention group. Validated 7-day point prevalent (PP) abstinence rates were 33.6% and 30.0% in the intervention versus SBP groups, respectively (OR 1.17, 95% CI 0.90 to 1.54) at 3 months and 29.2% versus 28.6% (OR 1.03, 95% CI 0.78 to 1.36) at 12 months post-screening. Subgroup analyses indicated a significant increase in 7-day PP abstinence at 3 months with the intervention in women (33.9% intervention, 23.1% SBP, OR 1.70, 95% CI 1.15 to 2.53) but not in men (33.3% intervention, 37.8% SBP, OR 0.82, 95% CI 0.57 to 1.19).

**Conclusion:**

Around one-third of study participants were abstinent from smoking at 3 months post-screening irrespective of study arm, but adding the personalised intervention did not increase quit rates. Further research is needed exploring possible sex differences in efficacy of personalised SC support. The high overall quit rate reinforces the value of SC support delivered alongside LCS.

**Trial registration number:**

ISRCRN 63825779 and NCT03750110.

**WHAT IS ALREADY KNOWN ON THIS TOPIC**

- Lung cancer screening with low-dose CT likely offers a teachable moment for smoking cessation. The efficacy of using heart and lung images captured as part of this process to support attempts to quit smoking has not yet been investigated.

**WHAT THIS STUDY ADDS**

- High-intensity stop-smoking support, co-located and integrated within a lung cancer screening programme results in long-term quitting in around one-third of study participants. There appears to be no overall impact of providing personalised heart and lung images as part of the stop-smoking intervention, but there is potentially benefit to be gained from such an approach amongst women.

**HOW THIS STUDY MIGHT AFFECT RESEARCH, PRACTICE OR POLICY**

- This study has shown the positive effect on quit outcomes that can be achieved by embedding an intensive stop-smoking intervention delivered by advisors specifically trained to work with this high-risk population, regardless of the addition of the personalised booklet. Commissioners of lung cancer screening programmes must consider the potential to capitalise on the opportunity offered by attendance at such programmes, with the potential to increase quitting and decrease health inequalities. Further evidence is needed to understand whether personalised interventions may be an efficacious approach.

## Introduction

 Smoking is the largest contributor to developing lung cancer, and smoking cessation (SC) is the most effective way to reduce lung cancer mortality.^1^ The US Preventive Service Task Force recommended screening in 2013,^2^ and other high and middle-income countries are at various stages of implementation. Following recommendation by the UK National Screening Committee in 2022,^3^ the UK Government announced the roll-out of lung cancer screening (LCS) in England in 2023,^4^ and the European Union has also published a plan for LCS implementation through the SOLACE programme.^5^ While the prevalence of screen-detected lung cancer from five UK LCS programmes averaged 2.2%,^6^ smoking prevalence among participants in LCS trials has been reported to be in the region of one-third to over half of all attendees.^7–10^ There is, therefore, a major opportunity to reduce future cancer risk by helping people who smoke to quit.

The effects of SC extend beyond lung cancer, both in terms of health benefits and cost-effectiveness. The 2020 Surgeon General report identified 11 additional cancers caused by smoking^11^; further, people who smoke are at risk of premature death and reduced quality of life due to chronic obstructive pulmonary disease, heart disease and stroke. Modelling from the USA has indicated that adding tobacco treatment to LCS programmes could decrease deaths by an additional 14% and increase the number of life years gained by up to 81%.^12^ Evidence has shown that LCS may be a key opportunity to inform participants of the harmful effects of smoking and increase their motivation to stop.^13 14^ Since lung cancer and other smoking-related comorbidities are far more prevalent among people experiencing socioeconomic deprivation,^15^ integrating SC support into LCS could also reduce health inequalities.

Current evidence suggests that SC rates in LCS trials are promising, with more intensive, personalised and multimodality interventions delivered by a clinician appearing to be most successful.^16^ However, further research is needed to understand optimal treatment types, timing and content to maximise the potential benefit.^1 16^ A recent systematic review assessing the impact of using medical images to motivate risk-reducing behaviours by increasing perceived threat suggested that such interventions are promising, but that further intervention trials are needed.^17^ Theory-informed interventions that simultaneously bolster beliefs in the individual’s ability to quit (self-efficacy beliefs) and that doing so will be beneficial (response efficacy beliefs) may minimise fearful, defensive and avoidant responses to receiving potentially threatening health information.^18^

The current study aims to assess the efficacy of a personalised stop-smoking intervention incorporating participants’ own heart and lung scan images and is designed to optimise behavioural responses to receiving personalised risk information by explaining the clinical importance of these findings with supportive communication to enhance personal salience, self-efficacy and response efficacy.

## Methods

Reporting conforms to Consolidated Standards of Reporting Trials guidelines (online supplemental document 1). The full study protocol and amendment log can be found in online supplemental document 2 and 3.

### Study design

The study is a blind at the point of randomisation, parallel group 1:1 trial conducted within the Yorkshire Lung Screening Trial (YLST) in Leeds, UK, according to the previously published protocol.^19^ In brief, YLST was a randomised controlled trial to evaluate invitation to community-based low dose CT (LDCT) screening for lung cancer versus usual care in a targeted population at risk.

### Participants and eligibility

Participants in YLST were aged 55–80, registered with a General Practitioner in the Leeds Clinical Commissioning Group area, with a smoking history recorded in medical records and considered eligible for LDCT screening for lung cancer.^20^ All individuals who attended the LDCT screening visit who had smoked within the last month, or had an exhaled carbon monoxide (CO) reading ≥6 ppm were offered immediate SC support. Those accepting ongoing stop-smoking support were eligible for inclusion in the Yorkshire Enhanced Stop Smoking (YESS) study and were approached regarding participation at their 4-week follow-up consultation with a smoking cessation practitioner (SCP). Any individual who did not engage with the SCP at the time of the YLST LDCT screening visit for any reason was not eligible for randomisation into YESS.

### Randomisation, allocation concealment and blinding

After the screening visit, but before consent was taken for participation in YESS at the 4-week follow-up consultation, all those who had accepted ongoing stop-smoking support were randomised by the trial manager to either continued standard best practice (SBP) or intervention using concealed allocation. The randomisation sequence (1:1 in permuted blocks of random size up to size 6) was generated using a computerised random-number generator, and participants were allocated sequentially, overseen by the University of Nottingham Clinical Trials Unit. The sequence of treatment allocations was concealed until interventions had all been assigned and recruitment and data collection were complete. Once primary and secondary data from both treatment groups were analysed by the trial statistician, the groups were unblinded to the Trial Management Group (TMG). In order to ensure concealment at the time of recruitment/consent, the personalised risk information booklet (further detail provided later)^21^ or a blank booklet was enclosed in a sealed envelope which was opened by the SCP following consent/data collection. Blinding of SCPs to study arm allocation prior to consent commenced 5 months into recruitment following review of processes at a Trial Steering Committee meeting. For the first 5 months, SCPs were aware of trial allocation at the time of consent/data collection.

### Procedures

Unless explicitly declined, all eligible YLST participants attended a consultation with a specialist SCP, trained to National Centre for Smoking Cessation and Training standards,^22^ co-located within the mobile screening unit. Support was provided in line with the National Institute for Health and Care Excellence best practice guidance at the time of the study^23^ comprising one session of behavioural support at the time of the screening visit and provision of pharmacotherapy (either as nicotine replacement therapy through delegated prescribing at the visit and/or a commercially available e-cigarette or arranging a GP prescription for varenicline or bupropion). An information sheet relating to the YESS study was provided to all individuals at the initial consultation, to be re-visited by the SCP at the 4-week follow-up consultation. Follow-up contact was either face-to-face or by telephone, typically weekly but flexible according to participant preference, for up to 12 weeks from the date of the screening visit. Where study SCP follow-up was not possible, or if the individual preferred, contact details were passed to the local NHS stop-smoking service (SSS) for referral into community services immediately following the screening visit.

Approximately 4 weeks after the screening visit, the SCP arranged a face-to-face visit with the individual either at home or a community location (according to their preference) to ascertain current smoking status (with CO validation in those reporting abstinence). Informed written consent for participation in the YESS study was given, and individuals were treated according to their allocated group, as described below. This time period ensured that YLST scan results had been reported and annotated images were available for the intervention group.

### SBP group

Continued pharmacotherapy/e-cigarettes and behavioural support was offered and arranged, as outlined above.

### Intervention group—personalised booklet with LDCT scan images

In addition to SBP and YLST standard feedback, participants were provided information about their scan findings in a personalised booklet (online supplemental figure 1). Where emphysema or coronary artery calcification (CAC) had been identified, appropriate images were incorporated into the booklet, accompanied by brief text to provide context and explanation, and standardised theory-informed scripted advice for consultations according to content to boost self-efficacy and response-efficacy. The intervention was co-developed with members of the public living in areas of high socioeconomic deprivation and with a smoking history who were involved in intervention development to ensure that the intervention met the needs of the target population, reported previously.^21^ The booklet also contained generic short-term and longer-term benefits of quitting derived from the Smoke-free NHS website (www.nhs.uk/smokefree/why-quit/what-happens-when-you-quit) and contact details for the YESS trial manager, the local NHS SSS, the YLST clinical team and the assigned SCP.

### COVID-19 pandemic

Participants attempting to quit at the time of lockdown were sent enough products to last them for their 12-week treatment period, and continued telephone support was provided by our SCPs; at 4-week follow-up, participants randomised to receive the intervention had the booklet described to them by the SCP over the telephone, and the booklet was posted to participants after the call. The SCP telephoned the patient a week later to answer any patient queries following receipt of the booklet. Advisors remained blinded to the treatment group until the point of sharing the leaflet as they would have previously when meeting participants face-to-face. CO validation of quit outcome was suspended from the point of lockdown, hence it was not possible to collect validation for one round at the 4-week follow-up stage and two rounds at the 3-month and 12-month stage; for these individuals, only self-reported smoking status was recorded as a validated reading after agreement from the Trial Steering Committee.

Following a reduction in lockdown measures on 18 May 2020, CO testing resumed on 20 May 2020, provided that the patient was not ‘shielding’ and did not have any COVID-19 symptoms.

### Outcomes

The primary outcome measure was 7-day point prevalent CO-validated SC 3 months after the screening visit in all participants enrolled in the YESS study. Secondary outcome measures were self-reported 7-day point prevalent SC at 3 months, self-reported and validated 7-day point prevalent SC at 12 months, self-reported and validated prolonged SC at 3 and 12 months and attempts to quit smoking at 3 and 12 months as pre-defined in the trial analysis plan.

### Statistical analysis

The power calculation, including its assumptions, and the statistical analysis plan agreed in advance with the Trial Steering Committee have been published within the trial protocol.^19^ It was calculated that 1040 consented individuals would provide 90% power to detect a 10% difference in quit rate between groups. Between-group differences in baseline characteristics were assessed by χ^2^ or t-tests, and the effect of the intervention was evaluated by comparing the outcomes between intervention and SBP groups using logistic regression. Results are presented in terms of the proportion achieving abstinence in the two groups, the risk difference and the OR with 95% CIs. The number of quit attempts undertaken since baseline was compared between groups using the Poisson regression model. We did not adjust for any baseline covariate as there was no difference between groups due to randomisation. Analyses were performed on an intention-to-treat basis. Where outcome data were missing, participants were assumed to be smoking.^24^ A likelihood ratio test was used to test for interaction by sex, Index of Multiple Deprivation (IMD), level of education, age and scan outcome with the effect of the intervention on abstinence. Within the intervention group, differences in quit rates according to booklet content were assessed using χ^2^ or t-tests. Sensitivity analyses were conducted to examine the impact of several factors on our analyses (as described in the protocol paper^19^). The impact of the change in consent procedures was explored by omitting from the analysis those consented prior to the change in the timing of consent, and the impact of the COVID-19 lockdown period on CO validation and face-to-face consultations was assessed by excluding all those with outcomes measured during lockdown. A micro-costing approach was used to estimate the mean cost of the intervention and the cost per quit. After undertaking the planned analyses, we generated a Bayes factor from the primary outcome. We used a conservative approach for estimation using a half-normal distribution, where the mode at 0 indicated no intervention effect and the SD equal to the expected effect size. The trial was registered with ISRCRN and clinicaltrials.gov and overseen by the TMG. An independent trials steering committee provided executive oversight and approved the statistical analysis plan before analysis was undertaken.

## Results

**Figure 1:**
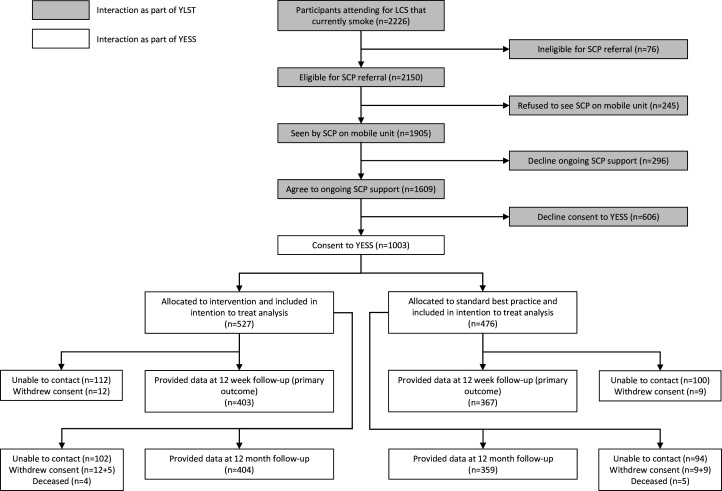
Participant flow and data collection through the YESS study. LCS, lung cancer screening; SCP, smoking cessation practitioner; YESS, Yorkshire Enhanced Stop Smoking study; YLST, Yorkshire Lung Screening Trial.

Participants were recruited between 10 December 2018 and 21 February 2021, with a suspension to recruitment between 7 March 2020 and 2 July 2020. Three-month and 12-month follow-up data collection was conducted between 18 February 2019 and 18 February 2022. In total, 1003 participants were randomly assigned to treatment groups (n=527 to intervention and n=476 to SBP) (figure 1). Loss to follow-up was 34.5% in the intervention arm and 36.8% in the control arm. Demographic and clinical characteristics of those completing the treatment course compared with those lost to follow-up are shown in online supplemental table 1. No meaningful differences were observed between completers and non-completers for any baseline characteristics, with the exception of IMD, which differed in both the intervention group (p=0.05) and the SBP group (p<0.0001). Participants in the SBP group completed an average of 6.2 consultations/interactions, compared with 6.7 in the intervention group (no significant difference). The majority were completed by telephone (74.2% vs 73.1%), on the mobile screening unit (14.7% vs 14.1%) and in the community (5.1% vs 5.8%).

Baseline characteristics were well balanced between trial arms (table 1). The mean age of participants was 65 (SD 7) years, half were men, and most were of white ethnicity. Most participants left school without formal qualifications (53.3%) and showed high levels of socioeconomic disadvantage, with 39.5% living in areas categorised within the lowest quintile of deprivation nationally. Participants most commonly had a dependency score indicating moderate addiction (44.8%) and a mean exhaled CO concentration of 16.4 (SD 10) ppm. Half of the participants indicated that they were extremely or moderately confident in their ability to quit and 29.0% self-reported 7-day point-prevalent abstinence from smoking on entry to the study (25.9% validated by CO measurement). There were no meaningful differences observed between those participants who completed or did not complete follow-up data collection for any baseline characteristics, with the exception of IMD quintile, with more of those completing data collection being in the most deprived quintile in both the intervention (p=0.05) and SBP group (p<0.00001) (online supplemental table 1).

**Table 1:** Participant characteristics by trial group* at baseline (entry into YLST)

	Total (n=1003)	Intervention (n=527)	SBP (n=476)
Age (years)			
Mean (SD)	65 (7)	65 (7)	65 (7)
Median (IQR)	65 (60–70)	64 (60–70)	65 (60–70)
Sex			
Male	504 (50.2)	279 (52.9)	225 (47.3)
Female	499 (49.8)	248 (47.1)	251 (52.7)
Ethnicity			
White	958 (96)	502 (95)	456 (96)
Black	17 (1.7)	11 (2.1)	6 (1.3)
Asian	14 (1.4)	7 (1.3)	7 (1.5)
Others	11 (1.1)	7 (1.3)	4 (0.8)
Unknown	3 (0.3)	0	3 (0.6)
IMD score, quintiles			
1 (most deprived)	396 (39.5)	191 (36.2)	205 (43.1)
2	170 (16.9)	82 (15.6)	88 (18.5)
3	175 (17.4)	106 (20.1)	69 (14.5)
4	172 (17.1)	95 (18.0)	77 (16.2)
5 (least deprived)	90 (9.0)	53 (10.1)	37 (7.8)
Level of education			
No qualifications	535 (53.3)	282 (53.5)	253 (53.2)
O-levels, CSE or GCSE	233 (23.2)	130 (24.7)	103 (21.6)
A-level and above	229 (22.8)	113 (21.4)	116 (24.4)
Unknown	6 (0.6)	2 (0.4)	4 (0.8)
Family history of lung cancer	193 (19.2)	97 (18.4)	96 (20.2)
CO reading (ppm)			
Mean (SD)	16.4 (10)	16.3 (9)	16.5 (10)
Median (IQR)	15 (10–21)	16 (10–21)	15 (10–21)
Unknown	2 (0.2)	0	2 (0.4)
Age started smoking (years)			
Mean (SD)	16.2 (4.1)	16.2 (4.3)	16.2 (3.8)
Median (IQR)	16 (14–18)	16 (14–18)	16 (14–18)
Unknown	2 (0.2)	0	2 (0.4)
Fagerstrom test of nicotine dependency			
<4 (less dependent)	345 (34.4)	178 (33.8)	167 (35.1)
4–6 (moderately dependent)	449 (44.8)	232 (44.0)	217 (45.6)
7–10 (highly dependent)	132 (13.2)	74 (14.0)	58 (12.2)
Unknown	77 (7.7)	43 (8.2)	34 (7.1)
Cigarettes per day			
≤10	338 (33.7)	163 (30.9)	175 (36.8)
11–20	484 (48.3)	271 (51.4)	213 (44.7)
21–30	114 (11.4)	58 (11.0)	56 (11.8)
>30	23 (2.3)	11 (2.1)	12 (2.5)
Unknown	44 (4.4)	24 (4.6)	20 (4.2)
Scan outcome			
Negative	769 (76.7)	424 (80.5)	372 (78.2)
Indeterminate	179 (17.8)	86 (16.3)	93 (19.5)
Positive	28 (2.8)	17 (3.2)	11 (2.3)
Motivation to quit smoking			
No intention/Intention to quit but don’t want to	294 (29.3)	148 (28.1)	146 (30.7)
Intention to quit but haven’t thought when	125 (12.5)	57 (10.8)	68 (14.3)
Intention to quit and hope soon	397 (39.6)	215 (40.8)	182 (38.2)
Intention to quit in the next 1–3 months	166 (16.6)	93 (17.6)	73 (15.3)
Already quit	11 (1.1)	6 (1.1)	5 (1.1)
Unknown	10 (1)	8 (1.5)	2 (0.4)
Times tried to quit the past year			
Mean (SD)	0.66 (2.1)	0.6 (1.5)	0.72 (2.6)
Median (IQR)	0 (0–1)	0 (0–1)	0 (0–1)
Unknown	5 (0.5)	3 (0.6)	2 (0.4)
Self-efficacy of quitting smoking			
How confident are you that you can quit?			
Extremely/moderately	500 (49.9)	267 (50/7)	233 (48.9)
Somewhat/slightly	337 (33.6)	178 (33.8)	159 (33.4)
Not at all	151 (15.1)	73 (13.9)	78 (16.4)
Unknown	15 (1.5)	9 (1.7)	6 (1.2)
7-day point prevalent abstinent			
4 weeks (validated)	260 (25.9)	137 (26.0)	123 (25.8)
4 weeks (self-reported)	291 (29.0)	155 (29.4)	136 (28.6)

Data are n (%), unless otherwise specified.

* No statistically significant differences between the two groups were detected.

CSE, Certificate of Secondary Education; GCSE, General Certificate of Secondary Education; IMD, Index of Multiple Deprivation; SBP, standard best practice; YLST, Yorkshire Lung Screening Trial.

CO validated 7-day point prevalent abstinence at 3 months was achieved by 177/527 (33.6%) of intervention participants and 143/476 (30.0%) in the SBP arm, a difference of 3.5% (95% CI −2.2% to 9.3%) (table 2). The intervention group had higher odds (OR=1.17; 95% CI 0.90 to 1.54) of abstinence but the difference was not statistically significant (Bayes factor 0.21). Among those participants who had quit smoking at the 4-week follow-up, there is no difference in CO-verified quit rates at 3 months between the intervention and SBP groups. The intervention was associated with higher quit rates among those who had not quit smoking at the 4-week follow-up, though the difference is not statistically significant (OR 1.34, 95% CI 0.95 to 1.89) (online supplemental table 2).

**Table 2:** Primary and secondary outcomes

Outcomes	Intervention (n=527)	SBP (n=476)	Risk difference % (95% CI)	Unadjusted OR (95% CI)	P value
*7-day point prevalent abstinence*					
3 months, validated*	177 (33.6)	143 (30.0)	3.54 (−2.21 to 9.30)	1.17 (0.90 to 1.54)	0.231
3 months, self-reported†	209 (39.7)	180 (37.8)	1.84 (−4.19 to 7.87)	1.08 (0.84 to 1.39)	0.554
12 months, validated†	154 (29.2)	136 (28.6)	0.65 (−4.97 to 6.27)	1.03 (0.78 to 1.36)	0.823
12 months, self-reported†	168 (31.9)	151 (31.7)	0.16 (−5.61 to 5.92)	1.01 (0.77 to 1.31)	0.966
*Prolonged smoking cessation*					
3 months, validated†	145 (27.5)	115 (24.2)	3.35 (−2.06 to 8.77)	1.19 (0.90 to 1.58)	0.295
3 months, self-reported†	165 (31.3)	141 (29.6)	1.68 (−4.01 to 7.38)	1.08 (0.83 to 1.42)	0.564
12 months, validated†	33 (6.3)	22 (4.6)	1.64 (−1.16 to 4.43)	1.38 (0.80 to 2.42)	0.258
12 months, self-reported†	44 (8.3)	33 (6.9)	1.41 (−1.87 to 4.70)	1.22 (0.77 to 1.97)	0.401
*Attempts to quit smoking*					
‡3-months†	–	–	–	0.94§ (0.82 to 1.08)	0.408
¶12-months†	–	–	–	1.08§ (0.92 to 1.26)	0.317

* Primary outcome.

† Secondary outcome.

‡ Data available for 770 participants.

§ The measure of effect is an unadjusted incidence rate ratio (IRR).

¶ Data available for 752 participants.

SBP, standard best practice.

Secondary outcomes were not statistically significantly different. At 12 months, 154/527 (29.2%) participants in the intervention and 136/476 (28.6%) in the SBP arm achieved validated 7-day point prevalent abstinence (OR=1.03; 95% CI 0.78 to 1.36). There was no difference in the number of contacts with SCPs between arms (mean 8.6 contacts intervention, 8.0 contacts SBP, p value 0.191). The average intervention cost was estimated at £175.1 (SD £77.5) per participant in the intervention arm and £124.0 (SD £76.8) in the SBP arm, with a cost per quitter at 3 months of £521.3 in the intervention group and £412.8 in the control group. The majority of the additional cost in the intervention arm was a result of staff costs for generating the booklet (£34.40 per participant)

There was a significant intervention effect in women (OR=1.70; 95% CI 1.15 to 2.53) but not in men (OR=0.82; 95% CI 0.57 to 1.19) (p_interaction_=0.002) for validated 7-day point-prevalent abstinence at 3 months (table 3). There was also a significant interaction effect by IMD quintile (though with no clear pattern across IMD categories) and a significant interaction with whether the scan outcome was intermediate/positive or negative (p_interaction_=0.038) but with no significant effect with either scan outcome. There was no modification in effect by age, education or type of pharmacotherapy used. There was also a significant effect modification by sex for validated 7-day point prevalence abstinence at 12-month follow-up (p=0.011) such that a significant effect of the intervention was still apparent in women, but not men (online supplemental table 3). Within the intervention group, there was no difference in quit rates according to the type of booklet provided (ie normal, CAC or emphysema) (table 4). Similar results were found after 12 months of follow-up (data not shown).

**Table 3:** Subgroup analysis of validated 7-day point prevalence abstinence after 3 months (primary outcome)

Outcomes	Intervention (n=527)	SBP (n=476)	Risk difference % (95% CI)	Unadjusted OR (95% CI)	P value (interaction)
Sex					0.008
Male	93 (33.3)	85 (37.8)	−4.44 (−12.85 to 3.96)	0.82 (0.57 to 1.19)	
Female	84 (33.9)	58 (23.1)	10.8 (2.89 to 18.63)	1.70 (1.15 to 2.53)	
Age (years)					0.388
55–60	51 (33.3)	37 (25.5)	7.81 (−2.48 to 18.11)	1.45 (0.88 to 2.42)	
61–65	41 (29.3)	26 (24.8)	4.52 (−6.66 to 15.70)	1.26 (0.71 to 2.25)	
66–70	39 (35.1)	33 (30.6)	4.58 (−7.84 to 17.00)	1.23 (0.70 to 2.17)	
71–80	46 (37.4)	47 (39.8)	−2.42 (−14.72 to 9.86)	0.90 (0.54 to 1.52)	
IMD					<0.001
1 (most deprived)	51 (26.7)	46 (22.4)	4.26 (−4.22 to 12.7)	1.25 (0.80 to 2.00)	
2	29 (35.4)	25 (28.4)	6.95 (−7.03 to 20.95)	1.38 (0.72 to 2.64)	
3	39 (36.8)	20 (29)	7.81 (−6.29 to 21.90)	1.42 (0.75 to 2.77)	
4	32 (33.7)	38 (49.4)	−15.60 (−30.33 to −1.00)	0.52 (0.28 to 0.96)	
5 (least deprived)	26 (49.1)	14 (37.8)	11.20 (−9.40 to 31.80)	1.58 (0.67 to 3.77)	
Level of education					0.776
No qualifications	88 (31.2)	70 (27.7)	3.53 (−4.18 to 11.30)	1.19 (0.82 to 1.72)	
O-levels, CSE or GCSE	47 (36.2)	35 (34)	2.17 (−10.20 to 14.50)	1.10 (0.64 to 1.90)	
A-level and above	42 (36.5)	37 (31.1)	5.42 (−6.67 to 17.50)	1.28 (0.74 to 2.20)	
Scan outcome					0.038
Negative	133 (31.4)	106 (28.5)	2.87 (−3.49 to 9.24)	1.15 (0.87 to 1.56)	
Indeterminate/positive	44 (42.7)	37 (35.6)	7.14 (−6.12 to 20.40)	1.35 (0.77 to 2.37)	
Pharmacotherapies and E-cigarettes					0.462
NRT	38 (26.8)	37 (33)	−6.27 (−17.60 to 5.07)	0.74 (0.43 to 1.27)	
E-cigarette	34 (37.4)	28 (29.5)	7.88 (−5.63 to 21.40)	1.42 (0.77 to 2.64)	
NRT+E-cigarette	61 (34.9)	45 (26.9)	7.91 (−1.84 to 17.70)	1.45 (0.91 to 2.31)	
Pharmacotherapy	20 (43.5)	16 (43.2)	0.20 (−21.20 to 21.70)	1.01 (0.42 to 2.43)	
None	22 (31.4)	16 (27.1)	4.31 (−11.40 to 20.00)	1.23 (0.58 to 1.67)	

IMD, Index of Multiple Deprivation; NRT, nicotine replacement therapy; SBP, standard best practice.

**Table 4:** The effect of booklet on validated 7-day point prevalence abstinence after 3 months (primary outcome) in the intervention group

	Total, n	Validated 7-day point prevalence abstinence after 3 months, n (%)	OR (95% CI)	P value
Normal heart	149	52 (29.3)	1.00	
Coronary calcification	378	97 (27.7)	0.92 (0.62 to 1.38)	0.689
Normal lung	135	43 (24.3)	1.00	
Any emphysema	392	92 (26.2)	1.11 (0.73 to 1.70)	0.621

A sensitivity analysis excluding those participants recruited before the change in consent procedure showed no impact on the primary outcome (online supplemental table 4) or any of the secondary outcomes (data not shown). Similarly, excluding those participants impacted by the COVID-19 pandemic showed no impact on the primary outcome (online supplemental table 5) or any secondary outcomes (data not shown).

## Discussion

This study, to our knowledge, is the first to assess the impact of using personalised heart and lung images highlighting CAC and emphysema captured during LDCT scanning as part of an LCS programme delivered as part of a comprehensive stop-smoking intervention. While there was no significant difference between intervention and SBP overall, around one-third of participants were CO validated as abstinent from smoking 3 months after the screening visit, and this quit rate was largely sustained at 12 month follow-up. Pre-specified subgroup analysis did show an increased quit rate in women but not men, although the study was not powered to look for sex differences in intervention effect. Study groups were well matched, and importantly, 4 in 10 people recruited were from the lowest quintile of deprivation.

This is the largest report to date of outcomes from a co-located SC service, and few studies to date have reported comparable data; notably, no trials have included a high intensity comparator arm. The findings are in line with a recent systematic review suggesting that high intensity, personalised and multimodality stop-smoking interventions are likely to be most effective.^16^ With nearly one-third of participants reporting abstinence from smoking at 3-month and 12-month follow-up, this study emphasises the need for, and benefits to be gained from integrating SC support as a routine component of LCS.

The QuLIT and QuLIT2 studies randomised people attending LCS as part of a Lung Health Check in London, UK, to either immediate access to SC support or usual care comprising very brief advice and signposting to existing services. QuLIT provided a face-to-face initial interaction,^25^ QuLIT2 used only telephone SC support due to the COVID-19 pandemic.^26^ Both studies reported quit rates of 21% in the intervention groups and 8%–9% in the control groups, hence also demonstrated a benefit to integrating stop-smoking support within an LCS programme. However, both studies also included participants not eligible for LCS (likely due to insufficient smoking history). In contrast, the current study was exclusively in participants eligible for LDCT screening and included a relatively high proportion from low socioeconomic groups, and so these may not represent directly comparable populations.

Despite the overall lack of effect of the personalised booklet and communication, there was a significant effect seen in female participants. Findings from the International Tobacco Control Four Country Survey reported that women were 31% less likely to quit smoking successfully than their male counterparts,^27^ and a later review of sex differences in SC in treatment studies reported that women appeared to be less likely to maintain longer-term SC.^28^ In the context of LCS, an analysis of 7369 participants in the US National Lung Screening Trial (NLST) who were smoking actively at enrolment showed lower quit attempts in women than men regardless of nicotine dependence level.^29^ Reflecting on this, 3 month quit rates in the SBP group in this study were considerably lower in women than men (23.1% vs 37.8%). However, there was no difference in the intervention arm (33.3% vs 33.9%).^29^ The finding that this study and a large retrospective analysis of NLST have demonstrated significant differences in SC behaviour according to sex suggests this may be a real effect. It is possible that the provision of the booklet and efficacy communication mitigates this sex-related difference and may benefit female participants in screening. However, this study was not powered to look for sex differences in intervention effect, and future research should explore this further. There was no overall difference in quit rates according to the presence of emphysema or CAC identified within the intervention booklet; however, similar to the point noted above, this study was not powered to look for these differences and may benefit from further exploration.

Strengths of the study include the collection of data on validated abstinence from smoking, outcome measures up to 12 months post screening visit and relatively low loss to follow-up among a sample with over-representation of socioeconomic disadvantage. In addition, we conducted a comprehensive and robust process evaluation to examine influences on the delivery of the intervention, including fidelity, exposure, contamination, context and theory testing, which will aid interpretation of intervention effects.^30^ The current study is subject to a number of limitations. First, being conducted in one city in the UK may limit generalisability to the wider population. However, attendance at LCS tends to be largely homogenous due to the eligibility criteria for screening regardless of setting and, crucially, includes those experiencing long-term and high tobacco dependence who find it more difficult to quit. The study population was predominantly white, limiting generalisability of the findings; however, this is reflective of the population eligible for and attending LCS within the geographical area in which the study took place. The results reported are from a trial setting and thus may be less applicable to a national LCS programme with fewer resources to invest in the same standard of stop-smoking support; however, they do illustrate what is achievable in this setting with appropriate investment.

The study has highlighted areas for future research; most notably the potential impact for a personalised intervention incorporating visual health information to support SC among women attending for LCS. However, the finding that the personalised booklet did not increase the quit rate over and above intensive standard stop-smoking support is important when considering future potential implementation and resource allocation. The study results clearly illustrate the positive quit outcomes that can be achieved by embedding an intensive stop-smoking intervention delivered by advisors specifically trained to work with this high-risk population, within a holistic and person-centred LCS programme, regardless of the addition of the personalised booklet. Commissioners of LCS programmes must consider the potential to capitalise on the opportunity offered by attendance at such programmes, with the potential to increase quitting and decrease health inequalities.

## Supplementary material

10.1136/thorax-2025-224217 online supplemental file 1

10.1136/thorax-2025-224217 online supplemental file 2

## Data Availability

Data are available upon reasonable request.
