# Supplementary material for: Randomised trial of delivering co-located, personalised stop-smoking support within lung cancer screening: the YESS study
Source: Thorax. 2026 Mar 13;81(9):e224217. doi: 10.1136/thorax-2025-224217 (PMC13479616; doi:10.1136/thorax-2025-224217)
Supplement: Supplementary file 1 [file thorax-81-9-s002.pdf]

Yorkshire Enhanced Stop Smoking Study (YESS) Personalised stop-smoking support within lung cancer screening

Murray RL et al. Thorax 2026. DOI: 10.1136/thorax-2026-224217

Population: 1003 people attending for lung cancer screening in Leeds, UK and reporting current smoking

Treatment: Both arms recieved behavioural counselling and NRT/E-cigarettes/medications

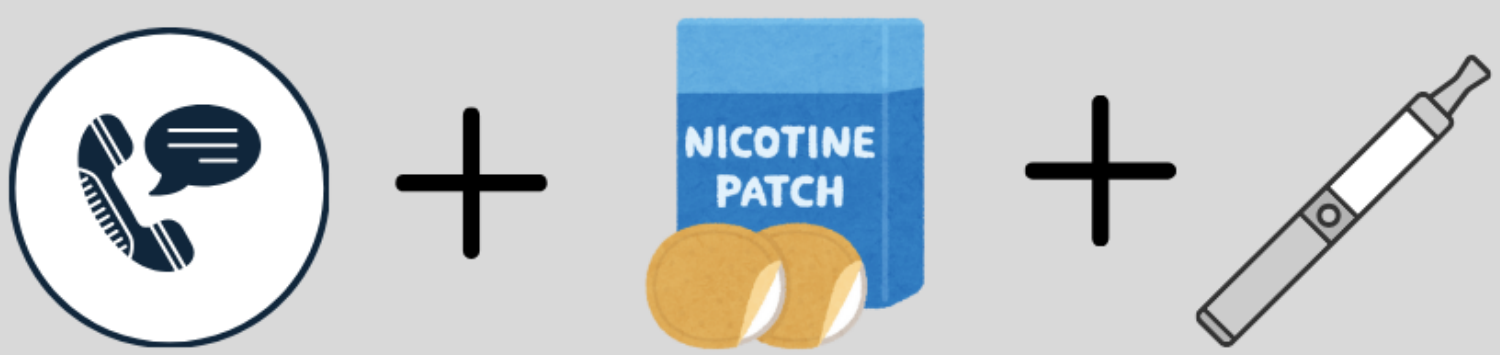

The Intervention group additionally received personalised booklets showing **their own** heart and lung images

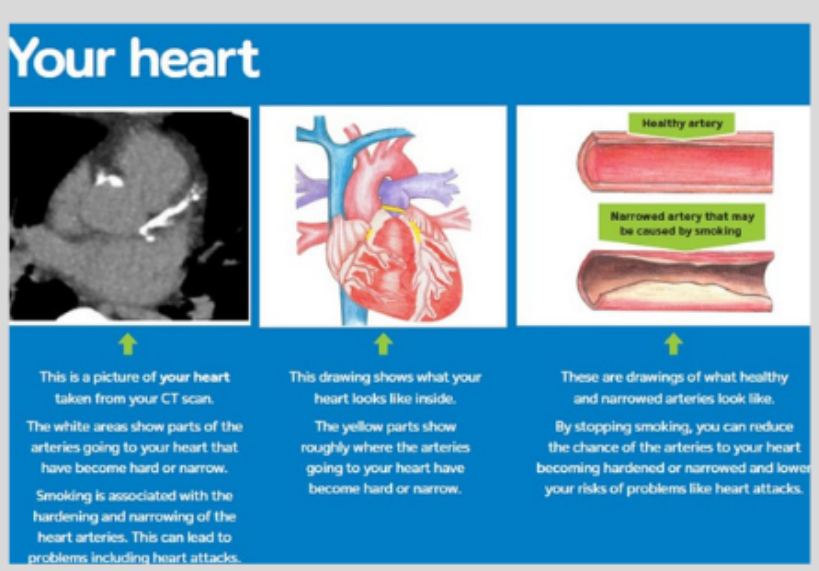

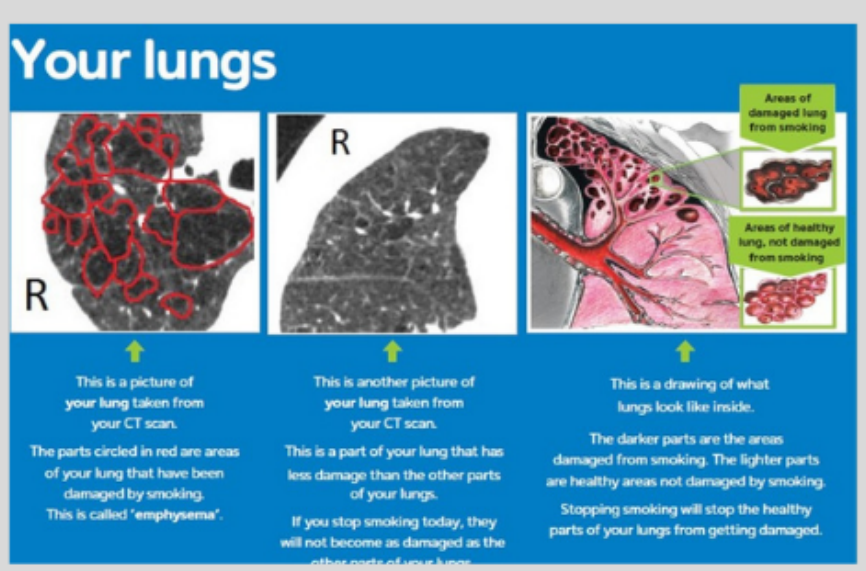

Outcome: Carbon Monoxide (CO) validated 7-day point prevalent abstinence at 3 months and 12 months.

7 Day PP Abstinence	Standard best practice (n=476) 47.5%	Intervention (n=527) 52.5%	Unadjusted OR (95% CI)
**3 months**	30.0%	33.6%	1.17 (0.90-1.54)
**12 months**	28.6%	29.2%	1.03 (0.78-1.36)
**Sex (3 months)**			
**Male**	37.8%	33.3%	0.82 (0.57-1.19)
**Female**	23.1%	33.9%	1.70 (1.15-2.53)

Conclusions

- Around 1 in 3 participants quit at 3 months.
- Personalised intervention did not improve overall quit rates
- Women may have benefited more from the intervention (further research is needed)
- Findings support integrating smoking cessation support within LCS

Thorax thorax.bmj.com @ThoraxBMJ British Thoracic Society BMJ Group
