# Supplementary material for: Randomised trial of delivering co-located, personalised stop-smoking support within lung cancer screening: the YESS study
Source: Thorax. 2026 Mar 13;81(9):e224217. doi: 10.1136/thorax-2025-224217 (PMC13479616; doi:10.1136/thorax-2025-224217)
Supplement: Supplementary file 2 [file thorax-81-9-s001.docx]

**Supplementary Materials**

**Title:** A randomised trial of delivering co-located, personalised stop smoking support within lung cancer screening: The YESS study

**Authors**

Rachael L. Murray, PhD¹*, David R. Baldwin, MD², Kate E. Brain, PhD³, John Britton, PhD¹, Christos V. Chalitsios, PhD¹, Philip A. J. Crosbie, PhD⁴, Sarah Lewis, PhD¹, Grace McCutchan, PhD³^,^⁵, Steve Parrott, MSc⁶, Samantha L. Quaife, PhD⁷, Rebecca Thorley, BA hons¹, Qi Wu, MSc⁶, Alexandra Ashurst, BHSc⁸, Rebecca J. Beeken, PhD⁹, Harriet Copeland, BSc⁹, Rhian Gabe, PhD¹⁰, Neil Hancock, BEng⁹, Catriona Marshall, PhD⁹, Richard D. Neal, PhD¹¹, Lucy R. E. Porter, MSc¹, Suzanne Rogerson, RN¹², Harriet D. Quinn-Scoggins, PhD³^,^¹³, Irene Simmonds, MA⁹, Alice Sloss^14^, Matthew E. J. Callister, PhD⁹^,^¹²

**Table of Contents**

| Supplementary material no. | Title | Page numbers |
| --- | --- | --- |
| 1 | **Supplementary Document 1:** CONSORT Checklist | 3-5 |
| 2 | **Supplementary Document 2:** Yorkshire enhanced Stop Smoking Study Protocol. | 6-38 |
| 3 | **Supplementary Document 3** Yorkshire enhanced Stop Smoking Study Protocol Amendment Log | 39-40 |
| 4 | **Supplementary Table 1**: Baseline characteristics of intervention and standard best practice study arms according to completion of follow-up data collection measures. | 41-42 |
| 5 | **Supplementary Table 2**: Quit status in those CO-verified abstinent from smoking according at 3-months according to quit status at 4-weeks | 43 |
| 6 | **Supplementary Table 3:** Subgroup analysis of validated 7-day point prevalence abstinence after 12-months | 44 |
| 7 | **Supplementary Table 4:** Primary and secondary outcomes by omitting from analysis those consented prior to the change in the timing of consent | 45 |
| 8 | **Supplementary Table 5:** Primary and secondary outcomes excluding participants with outcomes measured during COVID-19 lockdown period. | 46 |
| 9 | **Supplementary Figure 1**: Example personalised intervention booklet detailing scan findings of coronary artery calcification and/or emphysema. | 47 |

1. **Supplementary Document 1: Consort Checklist**

| **Section/topic** | **No** | **CONSORT 2025 checklist item description** | **Reported on page no.** |
| --- | --- | --- | --- |
| **Title and abstract** | | |  |
| Title and structured abstract | 1a | Identification as a randomised trial | 1 |
|  | 1b | Structured summary of the trial design, methods, results, and conclusions | 2 |
| **Open science** | | |  |
| Trial registration | 2 | Name of trial registry, identifying number (with URL) and date of registration | 3, 8,9 |
| Protocol and statistical analysis plan | 3 | Where the trial protocol and statistical analysis plan can be accessed |  |
| Data sharing | 4 | Where and how the individual de-identified participant data (including data dictionary), statistical code and any other materials can be accessed |  |
| Funding and conflicts of interest | 5a | Sources of funding and other support (eg, supply of drugs), and role of funders in the design, conduct, analysis and reporting of the trial | 17 |
|  | 5b | Financial and other conflicts of interest of the manuscript authors | 17 |
| **Introduction** | | |  |
| Background and rationale | 6 | Scientific background and rationale | 4, 5 |
| Objectives | 7 | Specific objectives related to benefits and harms | 5 |
| **Methods** | | |  |
| Patient and public involvement | 8 | Details of patient or public involvement in the design, conduct and reporting of the trial |  |
| Trial design | 9 | Description of trial design including type of trial (eg, parallel group, crossover), allocation ratio, and framework (eg, superiority, equivalence, non-inferiority, exploratory) | 5 |
| Changes to trial protocol | 10 | Important changes to the trial after it commenced including any outcomes or analyses that were not prespecified, with reason | 7,8 |
| Trial setting | 11 | Settings (eg, community, hospital) and locations (eg, countries, sites) where the trial was conducted | 5, 6 |
| Eligibility criteria | 12a | Eligibility criteria for participants | 5 |
|  | 12b | If applicable, eligibility criteria for sites and for individuals delivering the interventions (eg, surgeons, physiotherapists) | 5 |
| Intervention and comparator | 13 | Intervention and comparator with sufficient details to allow replication. If relevant, where additional materials describing the intervention and comparator (eg, intervention manual) can be accessed | 7 |
| Outcomes | 14 | Prespecified primary and secondary outcomes, including the specific measurement variable (eg, systolic blood pressure), analysis metric (eg, change from baseline, final value, time to event), method of aggregation (eg, median, proportion), and time point for each outcome | 8 |
| Harms | 15 | How harms were defined and assessed (eg, systematically, non-systematically) |  |
| Sample size | 16a | How sample size was determined, including all assumptions supporting the sample size calculation | 8 |
|  | 16b | Explanation of any interim analyses and stopping guidelines |  |
| Randomisation: |  |  |  |
| Sequence generation | 17a | Who generated the random allocation sequence and the method used | 5 |
|  | 17b | Type of randomisation and details of any restriction (eg, stratification, blocking and block size) | 5 |
| Allocation concealment mechanism | 18 | Mechanism used to implement the random allocation sequence (eg, central computer/telephone; sequentially numbered, opaque, sealed containers), describing any steps to conceal the sequence until interventions were assigned | 5 |
| Implementation | 19 | Whether the personnel who enrolled and those who assigned participants to the interventions had access to the random allocation sequence | 5, 6 |
| Blinding | 20a | Who was blinded after assignment to interventions (eg, participants, care providers, outcome assessors, data analysts) | 6 |
|  | 20b | If blinded, how blinding was achieved and description of the similarity of interventions | 6 |
| Statistical methods | 21a | Statistical methods used to compare groups for primary and secondary outcomes, including harms | 8,9 |
|  | 21b | Definition of who is included in each analysis (eg, all randomised participants), and in which group | 8,9 |
|  | 21c | How missing data were handled in the analysis | 8,9 |
|  | 21d | Methods for any additional analyses (eg, subgroup and sensitivity analyses), distinguishing prespecified from post hoc | 8,9 |
| **Results** | | |  |
| Participant flow, including flow diagram | 22a | For each group, the numbers of participants who were randomly assigned, received intended intervention, and were analysed for the primary outcome | 9 |
|  | 22b | For each group, losses and exclusions after randomisation, together with reasons | Figure 1 |
| Recruitment | 23a | Dates defining the periods of recruitment and follow-up for outcomes of benefits and harms | 9 |
|  | 23b | If relevant, why the trial ended or was stopped |  |
| Intervention and comparator delivery | 24a | Intervention and comparator as they were actually administered (eg, where appropriate, who delivered the intervention/comparator, how participants adhered, whether they were delivered as intended (fidelity)) |  |
|  | 24b | Concomitant care received during the trial for each group |  |
| Baseline data | 25 | A table showing baseline demographic and clinical characteristics for each group | 10, 11 |
| Numbers analysed,  outcomes and estimation | 26 | For each primary and secondary outcome, by group:  ● the number of participants included in the analysis  ● the number of participants with available data at the outcome time point  ● result for each group, and the estimated effect size and its precision (such as 95% confidence interval)  ● for binary outcomes, presentation of both absolute and relative effect size | 12, 13, 14  Supplementary file s41, s42 |
| Harms | 27 | All harms or unintended events in each group |  |
| Ancillary analyses | 28 | Any other analyses performed, including subgroup and sensitivity analyses, distinguishing pre-specified from post hoc | 14 |
| **Discussion** | | |  |
| Interpretation | 29 | Interpretation consistent with results, balancing benefits and harms, and considering other relevant evidence | 14, 15, 16 |
| Limitations | 30 | Trial limitations, addressing sources of potential bias, imprecision, generalisability, and, if relevant, multiplicity of analyses | 15, 16 |

Citation: Hopewell S, Chan AW, Collins GS, Hróbjartsson A, Moher D, Schulz KF, et al. CONSORT 2025 Statement: updated guideline for reporting randomised trials. BMJ. 2025; 388:e081123. <https://dx.doi.org/10.1136/bmj-2024-081123>
© 2025 Hopewell et al. This is an Open Access article distributed under the terms of the Creative Commons Attribution License (<https://creativecommons.org/licenses/by/4.0/>), which permits unrestricted use, distribution, and reproduction in any medium, provided the original work is properly cited.

*We strongly recommend reading this statement in conjunction with the CONSORT 2025 Explanation and Elaboration and/or the CONSORT 2025 Expanded Checklist for important clarifications on all the items. We also recommend reading relevant CONSORT extensions. See [www.consort-spirit.org](http://www.consort-spirit.org).

**2. Supplementary Document 2: Yorkshire Enhanced Stop Smoking Study Protocol.**

**The effect of adding a personalised smoking cessation intervention to a lung cancer screening programme**

**Version 2.4 16 07 2020**

**Short title:**	Yorkshire Enhanced Stop Smoking Study

**Acronym:**	YESS

**Trial Registration:**	[www.clinicaltrials.gov](http://www.clinicaltrials.gov) NCT03750110

**ISRCTN:**	63825779

**IRAS Project ID:**	240975

**Trial Sponsor:**	University of Nottingham

**Sponsor reference:**	18046

**Funding Source:**	Yorkshire Cancer Research (NOT414)

**SYNOPSIS**

| Title | The effect of adding a personalised smoking cessation intervention to a lung cancer screening programme (The Yorkshire Enhanced Stop Smoking Study) |
| --- | --- |
| Acronym | *YESS* |
| Short title | Yorkshire Enhanced Stop Smoking Study |
| Chief Investigator | Professor Rachael Murray |
| Objectives | This study aims to address a deficiency in the evidence base by comparing a theory based enhanced smoking cessation intervention with standard care for smokers who participate in the YLST. |
| Trial Configuration | The study will use a pragmatic, parallel-group, open, randomized controlled trial design |
| Setting | Delivered in the community as a complement to secondary care standard best practice (SBP) cessation package, in participants receiving the Lung Health Check as part of the Yorkshire Lung Screening Trial (YLST). |
| Sample size estimate | It is estimated that approximately 1083 people attending for screening over the two-year period will be eligible smokers. After excluding approximately 43 who attend during the initial 3-month trial development period, this leaves 1040 entering the definitive trial. With intention-to-treat analysis of all 1040 (520 per group) the study will have 90% power to detect a 10 percentage point higher quit rate at three months.. |
| Number of participants | Up to 1040 |
| Eligibility criteria | - Aged 55 to 80 years - Residing YLST study area. - Registered as a current or ex-smoker in a General Practice participating in YLST - Have attended ‘Lung Health Check’ as part of YLST, and agree to see the on-site Smoking Cessation Practitioner (SCP) - Smoked within the last month or have exhaled CO reading of 6ppm or above. - Have capacity to provide informed consent. |
| Description of interventions | **All participants:**  All individuals attending for a Lung Health Check who have smoked within the last month (eligible individuals) will be offered an immediate consultation with an SCP, trained to National Centre for Smoking Cessation and Training standard (40). The SCP will explain, advise and deliver smoking cessation support in accordance with NHS Stop Smoking Service best practice (and NICE PH48 guidance) over a four-week period. Individuals will be able to decline the offer of support should they so wish.  After the screening visit, but before the four-week follow up, all eligible individuals will be randomised to either standard best care or intervention.  At the four-week follow up visit (which will take place either at home, on the mobile unit, over the telephone if the patient is self-isolating due to suspected COVID-19 virus or social distancing or the community location according to patient preference) the SCP will collect written or verbal consent over the telephone for participation in the trial and if participants agree to take part they will be treated according to their pre-assigned treatment group.  **Usual care**  In accordance with current standard practice, smoking status will be assessed and validated in those reporting cessation by measuring exhaled CO. Participants who have successfully quit smoking at this point will be provided with continued pharmacotherapy/e-cigarettes and behavioural support in accordance with standard practice, and those who have relapsed to smoking encouraged to make another attempt to quit, with further behavioural support and pharmacotherapy as appropriate. All consenting participants will be contacted at three and twelve months after screening to ascertain current smoking status, with in-person visits for collection of exhaled CO samples arranged if abstinence is reported. If the patient is self –isolating or social distancing due to COVID-19 then the CO reading will be collected as soon as is practicable and safe to do so, but not longer than 4 weeks after the original data collection point. Pharmacotherapy/e-cigarettes and behavioural support will continue to be offered and arranged, as outlined above.    Those allocated to the intervention group will receive, in addition, the personalised feedback outlined below:  ***Intervention group***  At the four-week follow up visit, personalised feedback from the participants screening appointment will be provided.  The exact content of the intervention, and method of presentation is currently being refined but is likely to include:   - For participants with emphysema, an image of their own emphysematous lung compared to a library image of normal lung, or a section of their own non-emphysematous lung. Where emphysema is not present, library pictures showing normal and emphysematous lung will be used (see figure 1 below for example comparative images that may be provided). - For participants with coronary artery calcification, a cross sectional segment from their chest scan (either horizontal or vertical) showing the calcium compared to a library image of coronary arteries without calcification and/or a healthy section of the participant’s own heart scan. Where coronary artery calcification is not present, library pictures showing normal and calcified coronary arteries will be provided.   If patients are in self isolation or are social distancing related to COVID-19 consent into the study will be taken over the telephone. All of the 4 week procedures for each treatment group will be delivered over the telephone. If the patient is in the intervention treatment group the Smoking Cessation Practitioner (SCP) will, using the SCP script as a guide, describe the intervention leaflet to the patient, firstly by describing the lung images as showing some emphysema, or not showing emphysema, and the heart images as either showing or not showing calcification of the arteries. The SCP will then describe the short and long term benefits of smoking cessation and advise the patient where to get further help and support if required - as described in the leaflet. The SCP will then post the leaflet to the patient and call the patient in the next working week to answer any queries relating to the leaflet.  **Sub-section**  Those who decline to see the SCP, and those who decline to take part in the YESS study will be asked to complete a short screening questionnaire to explore their reasons for this decision,. Some will also be invited to take part in a sub-section of the main trial comprising of an interview (n = 30), and also asked to provide smoking status over the telephone (and CO validation in quitters) 3 and 12 months after screening. |
| Duration of study | Recruitment into the study will continue for 2 years, with an additional 12 months of follow up data collection from the point of the last participant is recruited. |
| Randomisation and blinding | The randomisation sequence (1:1 in permuted blocks of random size up to size 6) will be generated using a computer random number generator and patients allocated sequentially, and overseen by the University of Nottingham clinical trials unit. The trial interventions cannot be blinded but all analysis will be carried out blind to treatment allocation |
| Outcome measures | **Primary outcome**   - 7-day point prevalent CO validated smoking cessation three months after the Lung Health Check   **Secondary outcomes**   - 7-day point prevalent self-reported smoking cessation three months after the Lung Health Check Self-reported continuous smoking cessation at three months after the Lung Health Check - Self-reported continuous cessation at 12 months after the Lung Health Check - CO validated cessation at 12 months after the Lung Health Check - Self-reported continuous cessation at four weeks after the Lung Health Check - CO validated cessation at four weeks after the Lung Health Check - Changes in psychological variables, including perceived risk of lung cancer, motivation to quit smoking tobacco, confidence and efficacy beliefs (self and response) at all follow up points |
| Statistical methods | Primary analysis will be by intention-to-treat, including all those pre-randomised at Lung Health Check to usual care or intervention, and assuming, as usual in smoking cessation trials, that those who do not provide data on smoking status at four weeks, 3 months or 12 months, have continued to smoke.  We will compare descriptively the baseline characteristics of the two treatment groups and for those who consent, we will estimate the proportion who are retained in the study and provide data up to four weeks, three and 12 months and compare the characteristics of those who provide outcome data and those who do not. We will estimate the effect of the intervention by comparing our primary and secondary outcomes between intervention and standard best practice groups using multiple logistic regression, presenting results in terms of the % achieving abstinence in the two groups, the risk difference, and the odds ratio, with 95% confidence intervals. Primary analysis will assume that missing data implies smoking, but we will conduct sensitivity analysis to examine the impact on our findings of assuming alternative assumptions.  We will also assess the effect of adjusting for potentially important prognostic variables including lung cancer diagnosis and psychological variables. In addition to intention to treat analysis, we will conduct per protocol analysis including only those who consent to take part at four weeks; this will enable us to compare the effect of the enhanced intervention with usual care in those who agreed to receive it. We will compare cessation rates at four weeks between the intervention and standard best practice groups with those not eligible for the Lung Health Check, with those who refuse to see the SCP, those who refuse to take part in the trial and 55-80 year old community stop smoking service users to delineate the effect of both smoking cessation interventions within the YLST. All analysis will be conducted in Stata Version 14. |
| Process Evaluation | The trial process evaluation will include interviews, audio-recordings, SCP self-report and a questionnaire. Interviews will be conducted at four weeks, three and 12 months with trial participants across both intervention (n = 30) and standard best practice arms (n = 15). Intervention interviews will be used to explore intervention usage, acceptability and study contamination; standard best practice interviews will be used to explore potential study contamination. Interviews will also be conducted with SCPs (n = 4) across the three time-points (n = 12) to explore which aspects of the trial were perceived to influence change and gain an insight into contextual barriers and facilitators of embedding the intervention into practice. Audio-recordings of SCP sessions will take place (10% across each arm) to assess intervention fidelity and dose. To complement these, SCPs will be asked to self-report fidelity, dose and contextual information. |

**ABBREVIATIONS**

AE	Adverse Event

CCG	Clinical Commissioning Group

CI	Chief Investigator overall

CO	Carbon Monoxide

COPD	Chronic Obstructive Pulmonary Disease

CT	Computed Tomography

CRF	Case Report Form

CTU	Clinical Trials Unit

DMC	Data Monitoring Committee

GCP	Good Clinical Practice

GP	General Practitioner

GSL	General Sales Licence

HRA	Health Research Authority

ICF	Informed Consent Form

MPR	Multi Planar Reformation

MHRA	Medicines & Healthcare products Regulatory Agency

NCSCT	National Centre for Smoking Cessation and Training

NHS	National Health Service

NICE	National Institute for Heath and Care Excellence

NIHR	National Institute for Health Research

NLST	National Lung Screening Trial

NRT	Nicotine Replacement Therapy

LDCT	Low Dose Computed Topography (check)

LLP	Liverpool Lung Project

LTH	Leeds Teaching Hospital

PI	Principal Investigator at a local centre

PIS	Participant Information Sheet

PLCO	Prostate Lung Colorectal and Ovarian

QOF	Quality Outcomes Framework

REC	Research Ethics Committee

R&D	Research and Development department

SAE	Serious Adverse Event

SBP	Standard Best Practice

SCP	Smoking Cessation Practitioner

SSS	Stop Smoking Service

TMG	Trial Management Group

TSC	Trial Steering Committee

UK	United Kingdom

UKLS	United Kingdom Lung Screening

UKCTAS	UK Centre for Tobacco and Alcohol Studies

US	United States

USPSTF	United States Preventative Service Task Force

YCR	Yorkshire Cancer Research

YLST	Yorkshire Lung Screening Trial

**TABLE OF CONTENTS**

[**SYNOPSIS	3**](#_Toc516480753)

[**ABBREVIATIONS	7**](#_Toc516480754)

[**TRIAL / STUDY BACKGROUND INFORMATION AND RATIONALE	11**](#_Toc516480755)

[**TRIAL / STUDY OBJECTIVES AND PURPOSE	12**](#_Toc516480756)

[PURPOSE	13](#_Toc516480757)

[PRIMARY OBJECTIVE	13](#_Toc516480758)

[SECONDARY OBJECTIVES	13](#_Toc516480759)

[**DETAILS OF PRODUCT(S)	13**](#_Toc516480760)

[**Manufacture**	13](#_Toc516480761)

[**Packaging and labelling**	14](#_Toc516480762)

[**Storage, dispensing and return**	14](#_Toc516480763)

[**Placebo**	14](#_Toc516480764)

[**Known Side Effects**	14](#_Toc516480765)

[**TRIAL / STUDY DESIGN	15**](#_Toc516480766)

[TRIAL / STUDY CONFIGURATION	15](#_Toc516480767)

[Primary endpoint	15](#_Toc516480768)

[Secondary endpoint	15](#_Toc516480769)

[Safety endpoints	15](#_Toc516480770)

[RANDOMIZATION AND BLINDING	15](#_Toc516480771)

[Maintenance of randomisation codes and procedures for breaking code	16](#_Toc516480772)

[TRIAL/STUDY MANAGEMENT	16](#_Toc516480773)

[DURATION OF THE TRIAL / STUDY AND PARTICIPANT INVOLVEMENT	16](#_Toc516480774)

[End of the Trial	17](#_Toc516480775)

[SELECTION AND WITHDRAWAL OF PARTICIPANTS	17](#_Toc516480776)

[Recruitment	17](#_Toc516480777)

[Eligibility criteria	17](#_Toc516480778)

[Inclusion criteria	17](#_Toc516480779)

[Sub-Section	17](#_Toc516480780)

[Exclusion criteria	17](#_Toc516480781)

[Expected duration of participant participation	17](#_Toc516480782)

[Removal of participants from therapy or assessments/Participant Withdrawal	17](#_Toc516480783)

[Informed consent	18](#_Toc516480784)

[**TRIAL / STUDY TREATMENT AND REGIMEN	18**](#_Toc516480785)

[Standard best practice group	19](#_Toc516480786)

[Intervention group	19](#_Toc516480787)

[Generation of personalised risk information for intervention group	21](#_Toc516480788)

[Comparative measurement in those smokers attending community stop smoking services	23](#_Toc516480789)

[Comparative measurement in sub-section that opt out of participation in main trial	23](#_Toc516480790)

[Participants in Process Evaluation	23](#_Toc516480791)

[Compliance	23](#_Toc516480792)

[Criteria for terminating trial	23](#_Toc516480793)

[**Analyses	24**](#_Toc516480794)

[Methods	24](#_Toc516480795)

[Sample size and justification	26](#_Toc516480796)

[Assessment of efficacy	27](#_Toc516480797)

[Primary outcome	27](#_Toc516480798)

[Secondary outcomes	27](#_Toc516480799)

[Assessment of safety	28](#_Toc516480800)

[Procedures for missing, unused and spurious data	28](#_Toc516480801)

[Definition of populations analysed	28](#_Toc516480802)

[**ADVERSE EVENTS	28**](#_Toc516480803)

[**ETHICAL AND REGULATORY ASPECTS	28**](#_Toc516480804)

[ETHICS COMMITTEE AND REGULATORY APPROVALS	29](#_Toc516480805)

[INFORMED CONSENT AND PARTICIPANT INFORMATION	29](#_Toc516480806)

[RECORDS	29](#_Toc516480807)

[Case Report Forms	29](#_Toc516480808)

[Source documents	30](#_Toc516480809)

[Direct access to source data / documents	30](#_Toc516480810)

[DATA PROTECTION	30](#_Toc516480811)

[**QUALITY ASSURANCE & AUDIT	30**](#_Toc516480812)

[INSURANCE AND INDEMNITY	30](#_Toc516480813)

[TRIAL CONDUCT	30](#_Toc516480814)

[TRIAL DATA	31](#_Toc516480815)

[RECORD RETENTION AND ARCHIVING	31](#_Toc516480816)

[DISCONTINUATION OF THE TRIAL BY THE SPONSOR	31](#_Toc516480817)

[STATEMENT OF CONFIDENTIALITY	31](#_Toc516480818)

[**PUBLICATION AND DISSEMINATION POLICY	32**](#_Toc516480819)

[**USER AND PUBLIC INVOLVEMENT	32**](#_Toc516480820)

[**STUDY FINANCES	32**](#_Toc516480821)

[Funding source	32](#_Toc516480822)

[Participant stipends and payments	32](#_Toc516480823)

[**REFERENCES	33**](#_Toc516480825)

**TRIAL / STUDY BACKGROUND INFORMATION AND RATIONALE**

Lung cancer has the highest mortality of all cancers in the UK and is the single biggest cause of premature death in Leeds (1). It disproportionately affects those from lower socioeconomically deprived communities (2) where rates of smoking are highest (3). In 2014 in the Leeds South and East and the Leeds West Clinical Commissioning Group (CCG) areas, lung cancer standardized incidence rates were 131.4 and 103.3 respectively, compared to an average of 78.3 across England. Lung cancer standardized under-75 mortality rates were 52.4 and 39.3 respectively, compared to 33.26 across England (4). Poor outcomes from lung cancer treatment are typically the result of advanced, incurable disease at the time of symptomatic presentation. In 2011, the US National Lung Screening Trial (NLST) demonstrated a 20% reduction in lung cancer specific mortality, and a 6.7% reduction in all-cause mortality by annual low-dose CT (LDCT) screening, and lung cancer screening is now being adopted across North America having been recommended by the US Preventative Services Task Force (USPSTF) in 2013 (5). A number of European nations are deliberating introducing screening programmes for lung cancer, and await the results of the NELSON study (6), the only European study powered to demonstrate a mortality reduction, which remains in follow-up.

Despite widespread adoption in North America, several important questions remain regarding the best way to implement lung cancer screening. These include the optimum selection criteria to identify high-risk individuals to maximise clinical efficacy and cost-effectiveness, how to increase participation among ‘hard to reach’ populations, and how most effectively to embed smoking cessation support into these programmes (7, 8). The Yorkshire Lung Screening Trial, funded by Yorkshire Cancer Research, seeks to address the first two issues identified above, and is due to commence screening in 2018. However, there are no currently funded UK studies specifically investigating optimal smoking cessation provision within a lung cancer screening programme.

More than 85% of cases of lung cancer are caused by tobacco smoking (9), and stopping smoking, at any age, significantly reduces lung cancer risk (10, 11). It is therefore essential that any lung cancer screening programme provides smoking cessation support for participants, and doing so significantly increases the overall success of lung cancer screening programmes (12). One concern that has been raised around lung cancer screening for current smokers is the potential ‘moral hazard’ arising from a negative (i.e. reassuring) screening result, which may reduce motivation to quit (13). Conversely, attendance at a lung cancer screening programme offers a ‘teachable moment’ for smoking cessation, occurring at a time when participating smokers may be particularly receptive to offers of help to quit (14, 15) and indeed a negative screen result has been reported as being perceived as a ‘clean slate’ as a motivator to stop smoking (16). This observation fits with unpublished findings from a screening initiative in Manchester that despite a high prevalence of emphysema (68%) and coronary artery calcification (74%), most attendees were fit and relatively asymptomatic (85-90% performance status 0-1 and MRC dyspnoea scale 1-2), and hence potentially more susceptible to a message that lung damage had already occurred but clinical impact could be reduced by stopping smoking. One report from the NELSON study suggested a lower quit rate in screened persons compared to the unscreened control group, although a statistically significant effect did not persist following intention to treat analysis (17). No significant difference was seen between screened and control populations in the Danish Lung Cancer Screening Study (18), and the quit rate was significantly higher in screened versus control populations in the UK Lung Screening (UKLS) Pilot (19).

Evidence suggests that smoking cessation and LDCT screening have additive effects on survival. An analysis of participants in the NLST reported a 38% reduction in lung cancer mortality with the combination of smoking abstinence at 15 years with LDCT screening (20); in addition, detection of an abnormality on the CT scan was associated with increased likelihood of smoking cessation (21). Further analysis of the NLST showed that arranging the smoking cessation support at the time of the screening increased quit rates by 46% (although this intervention was delivered to only 10% of study participants) (22). In addition, research has indicated that adding smoking cessation interventions to LDCT screening improves the cost effectiveness of such programmes (23, 24)

However, while smoking cessation is clearly an effective preventive intervention there is little evidence on the design and optimization of the smoking cessation interventions and support used in the screening setting (25). Evidence from a systematic review published in 2014 showed a benefit of materials tailored to the characteristics of individual smokers (25), although the included studies were conducted predominantly in the general population, rather than screening participants. Less research has been conducted investigating approaches to cessation in smokers attending for lung cancer screening, although a recent study by Marshall and colleagues reported that it was feasible to deliver a single tailored session of motivational interviewing counselling on the day of screening (26).

For historical reasons NHS Stop Smoking Services (SSS) have evolved across the UK as opt-in facilities, whereby smokers who decide or agree to try to quit smoking then seek and are delivered help to do so. During the past five years however, evidence has grown that provision of smoking cessation support as an opt-out default generates far more quit attempts. We have demonstrated this to be the case in hospital inpatients, in which the opt-out model approximately doubled service uptake (27). Similar findings have been reported in relation to pregnant women setting quit dates in one NHS Foundation Trust, with uptake more than doubling after ‘opt out’ referrals were implemented (28). A study from the US found that 83% of eligible smokers did not opt out of a bedside consultation (29). Pro-active and systematic approaches to smoking cessation provision were recommended by NICE for pregnant women in 2010 (30) and across all acute, maternity and mental health settings in 2013 (31).

A recent UK study has also demonstrated the efficacy of including personalised risk information when inviting participants to smoking cessation services (32). A randomised controlled trial compared an individually tailored risk letter with an invitation to attend an introductory smoking cessation session to a standard generic letter advertising smoking cessation services. The personalised information was presented as a “Personal Health Risk Report”, and included information on the participant’s general health derived from Quality and Outcomes Framework (QOF) registered conditions on the GP record. In addition disease-specific health risks were included in the risk report, with the aim of making the individual aware of the personal health consequences of continuing to smoke, and their own individual risk of serious illness. The aim of providing this information was to change the individual’s balance of perceived ‘benefits’ against their understanding of the harm caused by smoking. The group receiving personalised risk information had significantly higher attendance at stop smoking service (17.4% vs. 9.0%, adjusted OR 2.2, 95% CI 1.8-2.7), higher completion of a 6-week NHS course (14.5% vs. 7.0%, adjusted OR 2.3, 95% CI 1.8-2.9) and higher 6-month validated abstinence (9.0% vs. 5.5%, adjusted OR 1.67, 95% CI 1.29-2.14).

To maximize smoking cessation among lung cancer screening participants it is therefore clearly important that all smokers who attend are provided with cessation support as a routine opt-out component of the screening visit, and that this support provides cessation pharmacotherapy and behavioural support in accordance with evidence-based NHS SSS practice. Furthermore the screening process, including risk assessment for lung cancer and imaging of the lung parenchyma and coronary arteries, allows an unprecedented opportunity for personalising the risks and harms of continued smoking, or conversely, the health gains to be made by successful smoking cessation. However there is currently no evidence on how best to design services to maximize the opportunity provided by screening participation, and how best to use the personalised information forthcoming from the screening process.

**TRIAL / STUDY OBJECTIVES AND PURPOSE**

This study aims to address the deficiency in the evidence base by comparing a theory based smoking cessation intervention with standard care for smokers who participate in the YLST.

**PURPOSE**

This study will test whether the provision of enhanced, personalised information, delivered by smoking cessation practitioners increases smoking cessation rates in the context of a lung cancer screening programme.

**PRIMARY OBJECTIVE**

Seven-day point prevalent CO validated smoking cessation three months after the Lung Health Check

**SECONDARY OBJECTIVES**

7-day point prevalent self-reported smoking cessation three months after the Lung Health Check Self-reported continuous smoking cessation at three months

•	Self-reported continuous cessation at 12 months

•	CO validated cessation at 12 months

•	Self-reported continuous cessation at four weeks

•	CO validated cessation at four weeks

•	Psychological variables, including perceived risk of lung cancer, motivation to quit smoking tobacco, confidence and efficacy beliefs (self and response)

**DETAILS OF PRODUCT(S)**

All participants will be offered free pharmacotherapy or e-cigarettes, according to participant preference, for as long as required by the participant (up to 12 weeks in total).

**Description**

In absence of specific contraindication, study participants will be recommended to use a transdermal patch (either the Nicotinell 21 mg 24-hour patch, or Nicorette Invisi Patch 15 mg or 25mg 16-hour patch dependent on tobacco dependency; the latter for individuals experiencing adverse effects from the 24 hour formulation) in conjunction with the Nicorette 15 mg inhalator. Alternative short-acting products offered will include the Nicorette nasal spray (500μg nicotine per spray), mouth spray (1mg nicotine per spray) or 4 mg gum. Participants in whom nicotine is contraindicated or who express a preference for an alternative to NRT will be offered varenicline (dose increased over 7 days to 1mg twice daily) or bupropion (dose increased to 150 mg twice daily), as recommended in the British National Formulary, after prescription from the participants’ general practitioner (GP) if deemed appropriate.

Electronic cigarettes will be provided directly to participants who wish to use them, in line with procedures employed by a number of stop smoking services including Nottingham and Leicester. Prior to study commencement, the research team will discuss purchasing preferences with reputable vape shops in the local study area in order to identify two variants that are popular with the local vape population and are included in MHRA notified products lists and these products will purchased and directly provided to participants at the point of the Lung Health Check. Both variants will be a third generation tank model, as these have been demonstrated to achieve greater nicotine delivery than the earlier ‘cigalike’ models (33).

**Manufacture**

NiQuitin products and bupropion are manufactured by Glaxo Smith Kline; Nicorette products by McNeil, and varenicline by Pfizer. All of the above products are licensed by the MHRA for use in promoting smoking cessation.

**E- Cigarettes**

Newer (second and third) generation e-cigarettes, often called personal vaporisers have a battery linked to a heating element or coil (atomiser) and a ‘tank’. The user fills the tank with their choice of e-liquid which may include flavours and contain nicotine in different concentrations (34) . The user takes a puff to activate the heating coil, which vapourises the e-liquid, creating a vapour that can be inhaled

The [Tobacco Products Directive 2014/14/EU (TPD)](http://ec.europa.eu/health/tobacco/docs/dir_201440_en.pdf) (35) introduced new rules for nicotine-containing electronic cigarettes and refill containers (Article 20) from May 2016 (36). The Medicines & Healthcare products Regulatory Agency (MRHA) is the competent authority for the notification scheme for e-cigarettes and refill containers in the UK and is responsible for implementing the majority of provisions under Article 20 (36).

From 20 May 2017, the new requirements include;

- Restricting e-cigarette tanks to a capacity of no more than 2ml.
- Restricting the maximum volume of nicotine-containing e-liquid for sale in one refill container to 10ml.
- Restricting e-liquids to a nicotine strength of no more than 20mg/ml.

In addition the MHRA require all e-cigarettes and e-liquids be notified to MHRA before they can be sold. Consumers and healthcare professionals can report side effects and safety concerns with e-cigarettes or refill containers to MHRA through the [Yellow Card](https://yellowcard.mhra.gov.uk/) reporting system (36).

The study will recommend third generation ‘tank’ e-cigarettes/ vaporisers in line with MRHA guidance and from the list of products already ‘notified’ to the MRHA.

**Packaging and labelling**

Products will be used in the manufacturer’s standard packaging and labelling, and for licensed medicines, dose instructions included in the packaging and manufacturers’ information sheets will be reiterated verbally to participants.

Electronic cigarettes will be provided to participants as packaged and labelled by the manufacturer; further verbal advice on use will be provided to individual participants in line with recommendations by NCSCT (34) by study staff as necessary and participants advised to contact supplying vape shops should they have any further technical queries.

**Storage, dispensing and return**

Nicotine Replacement products and e-cigarettes will be stored in a securely locked, fireproof cupboard, In the YLST office, below 25°C, only accessible to the Trial Manager, research assistant and the NCSCT (37) trained SCPs who will have clinical supervision provided by LTH. Sufficient supplies (i.e. quantities of each product which will last up to one week) will be kept on the mobile support truck in a lockable safe, to allow convenient access for the SCPs in initial consultations). Products will be dispensed by the study SCPs according to current LTH dispensing policy and practice. Surplus or unused stock will be returned to LTH to be added to their stock or destroyed in line with organisational policy for unused NRT.

**Placebo**

No placebos or other comparators will be used.

**Known Side Effects**

Documented side effects from the use of NRT include; Bloating; blurred vision; constipation; coughing; diarrhoea; dry mouth; dyspepsia; dysphagia; epistaxis; flatulence; gastritis; gastro-intestinal disturbances (may be caused by swallowed nicotine); hiccup; increased salivation; irritation of the throat; mild local reactions at the beginning of treatment are common because of the irritant effect of nicotine; minor skin irritation; mouth ulcers; nasal irritation; nausea; oesophagitis; paraesthesia; sneezing; vomiting; watery eyes (38) British National Formulary; Medicines Complete, Accessed February 2016, <https://www.medicinescomplete.com/mc/bnf/current/PHP3201-nicotine.htm#PHP46731-side-effects>.

Exposure to e-cigarettes appears to pose few risks, similar to oral NRT products, mouth and throat irritation are most commonly reported symptoms and these subside over time (34, 39).

**TRIAL / STUDY DESIGN**

**TRIAL / STUDY CONFIGURATION**

The study will use a pragmatic, parallel-group, open, randomized controlled trial design to test the effectiveness of an enhanced, personalised smoking cessation support package, tailored to include results from lung cancer screening, delivered as a complement to a standard best practice (SBP) cessation package, in participants in the Yorkshire Lung Screening Trial (YLST).

**Primary endpoint**

The primary outcome of this study will be 7-day point prevalent CO validated smoking cessation three months after the Lung Health Check

**Secondary endpoint**

The secondary outcomes are:

- 7-day point prevalent self-reported smoking cessation three months after the Lung Health Check
- Self-reported continuous smoking cessation at three months
- Self-reported continuous cessation at 12 months
- CO validated cessation at 12 months
- Self-reported continuous cessation at four weeks
- CO validated cessation at four weeks
- Psychological variables, including perceived risk of lung cancer, motivation to quit smoking tobacco, confidence and efficacy beliefs (self and response)

**Safety endpoints**

The study assesses the effect of personalised behavioural support using scan images and access to smoking cessation therapies, not the effectiveness of specific drug therapies. Any adverse effects or discontinuations reported during the trial will be recorded, and incidence compared between intervention and usual care groups. However, as the study assess the effect of the personalised behavioural support using scan images and access to smoking cessation therapies, not the effectiveness of untested drug therapies it is not anticipated that the study will require termination on safety grounds

**Stopping rules and discontinuation**

Participant recruitment is expected to end on 20th February 2021, with follow up data collection ending on 6th March 2022, in accordance with our funding limits. There are no other predefined rules for stopping or discontinuation

**RANDOMIZATION AND BLINDING**

All eligible participants will be allocated a unique identification number as part of the YLST which will be maintained through the YESS study. All will be randomized to either standard best practice or intervention groups using concealed allocation after attending for the lung health check, irrespective of whether they opt out of smoking cessation support, and in advance of being asked to provide informed consent following a Zelen’s study design (36) at the four-week follow up visit. This method of randomisation is commonly used in screening programmes (44) and it will allow us to assess the effect of introducing the personalised smoking cessation intervention into the lung cancer screening programme.

The randomisation sequence (1:1 in permuted blocks of random size up to size 6) will be generated using a computer random number generator and patients allocated sequentially, and overseen by the University of Nottingham clinical trials unit

Due to the nature of the intervention and how it will be delivered research staff, SCPs and participants will not be blinded to treatment group allocation. The trial statistician and the trial steering group will however remain blind to treatment allocation until the study analysis is complete.

No interim analyses of the data are planned and no stopping rules for the trial have been formulated.

**Maintenance of randomisation codes and procedures for breaking code**

The sequence of treatment allocations will be concealed until interventions have all been assigned and recruitment and data collection are complete. At the end of the study, when primary and secondary data from both treatment groups have been analysed by the trial statistician the groups will be un-blinded to the trial management group.

**TRIAL/STUDY MANAGEMENT**

This study represents a joint application between the Universities of Nottingham and Leeds. The Chief Investigator, from the UoN will lead the study and take overall responsibility for the running of the trial and delivery of the smoking cessation intervention. The Co-Investigator from the University of Leeds (LTH) will provide an essential liaison between the proposed trial and the YLST. The SCPs and radiographer will be managed by LTH, the research assistant by the University of Leeds.

A Trial Management Group (TMG) will oversee the day to day running of the trial providing clinical, scientific and practical advice and monitoring the progress of the trial regularly. An Independent Trials Steering Committee (TSC) will provide overall supervision and will include experts in cancer screening, respiratory medicine, smoking cessation and statistics. The TMG and TSC will meet prior to study commencement and then separately at regular intervals, to be agreed but approximately every six months.

The Chief Investigator has overall responsibility for the study and shall oversee all study management.

The data custodian will be the Chief Investigator.

**DURATION OF THE TRIAL / STUDY AND PARTICIPANT INVOLVEMENT**

Study Duration: Enrolment will align with the YLST, recruiting participants for two years. Participant recruitment is expected to end on 20th February 2021, with follow up data collection ending on 14th March 2022. The study will close 14th July 2022.

Participant Duration: Stop smoking support will be provided for as long as the participant requires, for up to 12 weeks. Follow up contact will be requested at 4 weeks, 3 months and 12 months, with a two week window to accommodate participant holidays/appointments extending to 4 weeks to accommodate collecting data from patients who are self-isolating or social distancing due to COVID-19. Where participants are invited for interviews as part of the process evaluation, these will take place within a two-week window where possible at 4 weeks, 3 months and 12 months.

**End of the Trial**

The end of the trial will be the last follow up of the last participant.

**SELECTION AND WITHDRAWAL OF PARTICIPANTS**

**Recruitment**

Participants included in this study will have been selected for screening and invitation to attend a lung health check as part of the Yorkshire Lung Screening Trial in line with their pre-defined inclusion criteria.

Those attending for a Lung Health Check in the first two years of the YLST programme who have smoked within the last month or have an exhaled Carbon Monoxide (CO) level of 6 ppm or above, and have the capacity to provide informed consent, will be eligible for inclusion in the current study. The nurse conducting the Lung Health Check will advise all participants meeting these criteria that after completing the screening process they will see a smoking cessation practitioner (SCP) co-located in the screening facility as part of the same appointment.

It will be explained to the potential participant that entry into the trial is entirely voluntary and that their treatment and care will not be affected by their decision. It will also be explained that they can withdraw at any time but attempts will be made to avoid this occurrence. In the event of their withdrawal it will be explained that their data collected so far cannot be erased and we will seek consent to use the data in the final analyses where appropriate.

**Eligibility criteria**

- Residing in the YLST study area in Leeds, UK
- Registered as a current or ex-smoker in a General Practice participating in YLST
- Have attended for a ‘Lung Health Check’ and agreed to see the SCP as part of YLST

**Inclusion criteria**

- Aged 55 to 80 years
- Smoked within the last month or have exhaled CO reading of 6ppm or above.
- Have capacity to provide informed consent.

**Sub-Section**

Participants who attend lung screening and either decline to see the SCP on the screening van or decline taking part in the main trial at the 4 week follow –up appointment with the SCP will be asked if they would like to take part in a sub-section of the main trial. It will be explained to the potential participant that entry into the trial is entirely voluntary and that their treatment and care will not be affected by their decision.

**Exclusion criteria**

All participants enrolled in the Lung Health Check will be eligible for inclusion in the study.

**Expected duration of participant participation**

Study participants will be participating in the study for a maximum of 12 weeks of active intervention, with the option to provide further consent for follow up visits at three and twelve months. Sub-section participants will ask to provide consent for follow-up at three and twelve months.

**Removal of participants from therapy or assessments/Participant Withdrawal**

Participants may be withdrawn from the trial either at their own request or at the discretion of the Investigator. The participants will be made aware that this will not affect their future care. Participants will be made aware (via the information sheet and consent form) that should they withdraw the data collected to date cannot be erased and may still be used in the final analysis.

**Informed consent**

All participants will provide written informed consent, except where patients are self-isolating or social distancing due to COVID-19, then consent will be taken over the telephone. The Informed Consent Form will be signed and dated by the participant before they enter the trial either in person on by post.. The Investigator will explain the details of the trial and provide a Participant Information Sheet, ensuring that the participant has sufficient time to consider participating or not. The Investigator will answer any questions that the participant has concerning study participation.

Informed consent will be collected from each participant before they undergo any interventions (including physical examination and history taking) related to the study. One copy of this will be kept by the participant, one will be kept by the Investigator.

Should there be any subsequent amendment to the final protocol, which might affect a participant’s participation in the trial, continuing consent will be obtained using an amended Consent form which will be signed by the participant.

**TRIAL / STUDY TREATMENT AND REGIMEN**

***In Summary***

We will commence trial recruitment as soon as the YLST trial commences (current anticipated start date 02/10.2018), thus ensuring that all YLST participants who smoke are offered standard best care. Development work as to the content of the intervention has already been conducted by Cardiff University, however, cognitive testing with a small sample of intervention users will be carried out to assess understanding and acceptability of personalised risk information, and to further refine the tailored feedback materials, presenting information that is perceived to be salient and effective by the Yorkshire population. Qualitative process interviews will commence with around ten participants at the start of the screening trial to assess intervention feasibility and optimise design. 'SCPs will receive standardised training on how to deliver the personalised intervention to ensure that it is delivered consistently. This will include face-to-face training, example scripts of conversations and clinical training as to how to discuss the scan images provided by clinical co-applicants. SCPs will be observed during their first six sessions.

***Trial Regimen***

Standard Best Practice at initial Lung Health Check; delivered to all individuals who accept the offer of support

There is currently no standard of ‘usual care’ for smoking cessation delivery within lung cancer screening programmes. However, delivery of stop smoking support in lie with NICE guidance for NHS settings will be offered to all eligible participants. Immediately after being screened as part of the YLST, the on-site SCP (employed by Leeds Teaching Hospitals and supported by the YLST clinical team) will provide standard smoking cessation support in line with NICE PH48 guidance on the same van as they will receive the lung health check, comprising one session of behavioural support and provision of pharmacotherapy (either as Nicotine Replacement Therapy (NRT) through delegated prescribing at the time of the visit or arrange a prescription for varenicline or bupropion via the participant’s General Practitioner), and follow-up contact either face-to-face or by telephone, typically weekly but more or less frequently according to participant preference.

In addition, a commercially available e-cigarette will be offered to participants who express a preference for their use. Whilst not currently provided as a smoking cessation aid on the NHS, guidance on e-cigarette use for smoking cessation produced by the National Centre for Smoking Cessation and Training recommends that services support users who choose to use e-cigarettes in their quit attempts (34), and a number of stop smoking services (for example Leicester and Nottingham City) are providing e-cigarettes and liquids directly to clients for this purpose. Further, e-cigarettes and liquids are being directly provided to participants as part of a number of research studies around the UK. Use of electronic cigarettes in conjunction with NRT patches, or as a single therapy, will be encouraged for participants expressing this preference.

NRT will typically comprise combination therapy with transdermal patch and short-acting formulation, used for as long as the participant finds them helpful (typically 6-9 weeks); and varenicline and bupropion for their standard licensed periods of up to 9 weeks. Participants who prefer to use their own electronic cigarette as a short-acting NRT substitute will be encouraged to do so. On-going smoking cessation support will be provided by the SCP for as long and as often as the smoker accepts for a period of four weeks; typically via telephone but with face-to-face meetings where feasible. Where SCP follow up is not possible, or if the individual smoker prefers, contact details will be passed to the local SSS for referral into community services immediately following the screening visit.. Any eligible participant who opts out of smoking cessation support will be given, and verbally encouraged to use, contact details for local NHS Stop Smoking Services. All eligible participants (including those who decline the offer of a visit to the SCP) will be asked by research nurses employed by the YLST for consent to be contacted by this study over the telephone at four weeks to ascertain smoking status with optional CO validation if quit.

After the screening visit, but before the four-week follow up, all eligible individuals will be randomised to either standard best care or intervention.

Approximately four weeks after the screening visit (and hence after scan results have been issued by YLST), the SCP will arrange a face to face visit with the smoker either at home, on the mobile unit or community location (according to the participant’s preference) to ascertain current smoking status (with CO validation in those reporting abstinence). Where the patient is self-isolating or social distancing due to COVID-19 the SCP will make arrangements and carry out this meeting over the telephone. Informed written consent for participation in the trial will be confirmed. If participation in the trial is confirmed the individual will then be treated according to the pre-assigned treatment group and as described below. Smokers who do not wish to take part in the study will be asked for consent to be contacted by the study team to ascertain smoking status in the future, and some may be invited for interview as part of the process evaluation described later. Those who decline the study but wish to continue with cessation support will be referred to their LSSS.

**Standard best practice group**

Pharmacotherapy/e-cigarettes and behavioural support will continue to be offered and arranged, as outlined above. All consenting participants will be contacted at three and twelve months to ascertain current smoking status, with in-person visits for collection of exhaled CO samples arranged if abstinence is reported.

**Intervention group**

As a supplement to standard best practice and YLST standard feedback, 4 weeks after the lung health check, the SCP will arrange a face to face visit with the individual (either at home, on the mobile unit or community location according to the participant’s preference) to ascertain current smoking status (with CO validation in those reporting abstinence) and provide the personalised enhanced intervention where personalised feedback from their screening appointment will be provided. Exact details of the enhanced intervention are still being developed and refined in conjunction with patient and public involvement groups, but will most likely include:

- For participants with emphysema, an image of their own emphysematous lung compared to a library image of normal lung, or a section of their own non-emphysematous lung. Where emphysema is not present, library pictures showing normal and emphysematous lung will be used (see figure 1 below for an example comparative image that may be provided). Data from the Manchester Macmillan CT screening pilot shows that 68% of screening participants had emphysema on CT scans.
- For participants with coronary artery calcification, a cross sectional segment from their chest scan (either horizontal or vertical) showing the calcium compared to a library image of coronary arteries without calcification. Where coronary artery calcification is not present, library pictures showing normal and calcified coronary arteries will be provided. Data from the Manchester Macmillan CT screening pilot showed 74% of screening participants had some degree of coronary artery calcification.

**Figure 1*: Example comparative images* showing (clockwise from top) areas of the lung damaged by smoking, healthy areas of the lung and an artist’s impression of a lung illustrating damaged and healthy areas.**

The SCP will explain the biological nature of emphysema and coronary artery calcification (suggestive of coronary artery narrowing and linked with increased risk of cardiovascular events), and will provide feedback and further smoking cessation advice tailored to the result. The SCP will highlight specific benefits to risks of lung cancer, COPD or cardiovascular disease arising from quitting. We have consciously decided not to highlight the presence of indeterminate nodules in the cessation information provided. This will be fed back to the participant as part of the YLST, but given that the malignant risk of nodules entering surveillance is low (approximately 2%), the overall message from the main screening study is one of reassurance that these nodules are likely to be benign. Therefore using the presence of these nodules to support smoking cessation would not be appropriate. All consenting participants will be contacted at three and twelve months to ascertain current smoking status, with in-person visits for collection of exhaled CO samples arranged if abstinence is reported.

Participants in the intervention will be offered the above package irrespective of the outcome of the CT scan. However, for those participants with positive scans (who will have been referred to Lung Cancer Fast-Track clinic), the importance and benefits of quitting smoking to the natural history of treatment for lung cancer will be enhanced, and participants encouraged to continue in the cessation programme. The path of individuals through the study is illustrated in Figure 2.

If patients are in self-isolation or social distancing due to COVID-19 consent into the study will be taken over the telephone. All of the 4 week procedures for each treatment group will be delivered over the telephone. If the patient is in the intervention treatment group the Smoking Cessation Practitioner (SCP) will, using the SCP script as a guide, describe the intervention leaflet to the patient, firstly by describing the lung images as showing some emphysema, or not showing emphysema, and the heart images as either showing or not showing calcification of the arteries. The SCP will then describe the short and long term benefits of smoking cessation and advise the patient where to get further help and support if required - as described in the leaflet. The SCP will then post the leaflet to the patient and call the patient in the next working week to answer any queries relating to the leaflet.

**Generation of personalised risk information for intervention group**

Prior to the four week visit for the intervention group, a pamphlet will be produced comprising personalised risk information including participants’ own scan images.

**Figure 2:** **Flow diagram illustrating the path of participants through the study, interventions and evaluations.**

Where the CT screen shows emphysema or coronary artery calcification (estimated prevalence 68% and 74% respectively from Manchester Macmillan CT screening pilot data), images will be extracted from the scan by the radiographer to be included in the pamphlet.

The presence or absence of emphysema and coronary artery calcification would be indicated in the radiology report that will be generated in YLST. A trained research radiographer would identify the cross-sectional CT image on lung windows displaying the most significant emphysema, and this will be displayed alongside a library picture showing normal lung parenchyma and/or a healthy section of the participant’s own lung. Coronary artery calcification will be displayed as a cross sectional segment from the chest scan (either horizontal or vertical) showing the calcium. Again the participant’s image will be displayed alongside a library picture showing normal non-calcified coronary arteries and/or a healthy section of the participant’s own heart scan. For the 32% and 26% of patients who will have no emphysema or coronary artery calcification respectively, library images showing both features alongside normal scans will be shown to illustrate the possible effects of continuing smoking.

**Comparative measurement in those smokers attending community stop smoking services**

Anonymised data will be provided for those aged 55-80 years for dates corresponding to the study period from the Leeds local stop smoking service to compare quit outcomes in those receiving smoking cessation support through YESS with those accessing community services.

**Comparative measurement in sub-section that opt out of participation in main trial**

Those who decline to see the SCP on the screening van, and those who decline to take part in the YESS study will be asked to complete a short screening questionnaire to explore their reasons for declining. Some will also be invited to take part in a sub-section of the main trial comprising of an interview (n = 30), and also asked to provide smoking status over the telephone (and CO validation in quitters) 3 and 12 months after screening.

**Participants in Process Evaluation**

A mixed-methods process evaluation will be undertaken to gather information that will aid interpretation of trial effects in light of knowledge gained about intervention fidelity (delivered as intended) and dose (quantity of intervention delivered) (52).

Qualitative interviews will be undertaken with participants and SCPs (approximately 57 in total). Interview data will be collected and analysed iteratively to allow themes interpreted in early interviews (4 weeks and 3 months) to be developed and explored in later interviews (at 12 months) (52).

**Compliance**

Compliance will be measured in relation to acceptance of usual care cessation support reported by participants in the usual care group at follow-up visits, and intervention components delivered to those in the intervention group. Non-smoking status will be validated objectively using exhaled CO. There will be no defined level of acceptable compliance however and data from all consenting participants will be included in the analysis with the assumption that those in whom validated abstinence is not confirmed by and exhaled CO < 6ppm have relapsed to smoking.

**Criteria for terminating trial**

Recruitment to the trial is time limited since all participants will be recruited as part of the first round of the YLST, which will complete by September 2020. There are no other termination criteria

**Analyses**

**Methods**

**Process evaluation**

Interviews will include approximately 30 semi-structured interviews (10 at each time point) with intervention participants, or until data saturation, to evaluate intervention usage, comprehension, and acceptability. We will also interview 15 control group participants (5 at each time point) to assess potential study contamination. The interviews will also be used to explore whether participants received any additional risk information or smoking cessation advice from health care professionals as part of their lung health check or during any nodule follow-up.

We will purposively sample participants by age (≤65 years, >65 years), gender (male/female), quit motivation where possible (as indicated at the initial consultation) and level of lung damage across all interview time-points. At 3 and 12 month interviews participants will be additionally sampled according to those who have and have not quit. Interviews will be carried out face-to-face or by telephone, according to preference, and participants will be offered a shopping voucher. Where possible interviews will be conducted within a two-week window after study follow-up data collection. We will conduct interviews across the two year trial recruitment timeframe.

Interviews with SCPs (n = 4) will also be performed across the three time points (4 weeks, 3 months and 12 months), at a total of twelve interviews. Interviews with SCPs will explore which aspects were perceived to influence change, gain insight into contextual barriers and facilitators of embedding the personalised intervention into practice and intervention fidelity.

All interviews will be audio-recorded using an encrypted recorder with permission, transcribed verbatim, and the data anonymised. The encrypted audio recording will be labelled using a trial number and emailed to an external transcribing service, who will ‘type-up’ the content of the interview and then return it to us via e-mail. Audio- files will be destroyed at the end of the trial and after analysis is complete.

Audio-recordings of SCP sessions will take place (10% across both arms) to assess intervention fidelity. Sessions to be recorded will be purposefully sampled by SCP, quit motivation (where possible), age (≤65 years, >65 years) and gender (male/female). We will conduct audio-recordings across the two year trial recruitment timeframe. Fidelity will be ascertained by a trained researcher listening to the audio-recordings and coding the appropriate delivery of materials against standard care (control) and standard care plus personalised risk information (intervention). A standardised assessment proforma will be developed and used, and a fidelity definition and acceptable range will be agreed upon in advance by the team. Coding will be conducted primarily by one researcher with independent double-coding performed for 20% of all recordings (10% of recordings across both arms). To complement session audio-recordings and SCP interviews, SCPs will be asked to self-report fidelity (intervention elements were delivered as intended) and dose (how much of the intervention was delivered). All interviews will be audio-recorded using an encrypted recorder with permission, transcribed verbatim, and the data anonymised. The encrypted audio recording will be labelled using a study number and emailed to an external transcribing service, who will ‘type-up’ the content of the interview and then return it to us via e-mail. Audio- files will be destroyed at the end of the trial and after analysis is complete.

As an addition to the short questionnaire completed by study decliners we will again purposively sample approximately 30 people by quit motivation (where possible), age (≤65 years, >65 years) and gender (male/female) for interview. Interviews will be carried out face-to-face or by telephone, according to preference, and participants will be offered a shopping voucher. Where possible interviews will be conducted within a two-week window after declining. We will conduct interviews across the two year trial recruitment timeframe. Consent for interviews with those that decline will be obtained via telephone according to a standardised consent script. Information sheets and a copy of the completed consent form will be sent to participants.

***Economic evaluation***

We will undertake an economic evaluation alongside the trial to assess the cost-effectiveness of the personalised feedback intervention over and above standard.  An NHS perspective will be adopted in line with NICE guidance.  Cost-effectiveness evidence is important when making a case for the adoption of an intervention into routine practice, in order to demonstrate that the programme represents value for money. We will record and calculate the costs of providing both standard care and the intervention prospectively throughout the trial, in terms of staff time, overheads, pharmacotherapy and other consumables.  We will also record patients’ utilisation of primary and secondary health care services outside of the trial using a short self-report questionnaire.  The questionnaire will be administered at baseline and 12 month follow-up as part of the assessment battery.  Costs of wider health care will be calculated using national average unit costs.  A total health care cost profile for all patients in each trial arm is then constructed by adding the costs of the intervention and standard care to the wider NHS costs. The economic evaluation will use validated quit rates at 12 months to estimate the incremental cost per quitter afforded by the intervention over and above standard best practice.   The results presented will indicate the NHS cost of funding the intervention in order to gain one additional quitter at 12 months.

**Analysis**

Primary analysis will be by intention-to-treat, including all those pre-randomised at Lung Health Check to usual care or intervention, and assuming, as usual in smoking cessation trials, that those who do not provide data on smoking status at 4 weeks, 3 months or 12 months, have continued to smoke. We will compare descriptively the baseline characteristics of the two treatment groups. We will estimate the proportion consenting to take part in the study (with a 95% confidence interval) at four weeks, and compare these proportions between treatment groups and according to baseline characteristics. For those who consent, we will estimate the proportion who are retained in the study and provide data up to three, 12 and 24 months and compare the characteristics of those who provide outcome data and those who do not. We will estimate the effect of the intervention by comparing our primary and secondary outcomes between intervention and standard best practice groups using multiple logistic regression, presenting results in terms of the % achieving abstinence in the two groups, the risk difference, and the odds ratio, with 95% confidence intervals. Primary analysis will assume that missing data implies smoking, but we will conduct sensitivity analysis to examine the impact on our findings of assuming alternative assumptions, including the Hedeker approach which assumes alternative relationships between having missing data and smoking, and using multiple imputation to test the alternative assumption that the data are missing at random (i.e. missing data can be modelled using measured variables). In secondary analysis, we will also assess the effect of adjusting for potentially important prognostic variables including lung cancer diagnosis and psychological variables as outlined in the secondary outcomes above. In addition to intention to treat analysis, we will conduct per protocol analysis including only those who consent to take part at 4 weeks; this will enable us to compare the effect of the enhanced intervention with usual care in those who agreed to receive it. We will compare cessation rates at 4 weeks between the intervention and standard best practice groups with those not eligible for the Lung Health Check to delineate the effect of both smoking cessation interventions within the YLST. Anonymised data on 55-80 year old service users accessing the local stop smoking service will be provided to allow comparison of characteristics and quit outcomes between smokers within YESS and those accessing community services for the same time period. Analysis will be conducted in Stata Version 14 on UoN computers and backed up to the UoN servers.

**Process Evaluation**

The process evaluation will be reported in line with EQUATOR guidelines. (53) All process evaluation data will be analysed on Cardiff University password protected computers and backed up to the Cardiff University servers.

***Interviews***

Anonymised interview transcripts will be analysed using a Framework approach (54). Using NVivo software or Microsoft Excel where appropriate for indexing, themes will be derived initially from the process evaluation aims and topic guides, with additional themes emerging from the data. The analytic framework will be refined by double coding 20% of the interviews (55). Analysis of participant, those that decline and provider interviews will be conducted separately, with comparisons made between the themes identified within the respective analyses.

***Audio Recordings***

Audio-recordings will be analysed for fidelity. Fidelity will be assessed through the coding of intervention components delivered against those pre-set for standard care or standard care plus personalised risk information. Coding will take place on a standardised preform (to be developed prior to study commencement), and a fidelity definition and acceptable range will be agreed upon in advance by the team. Twenty per cent of audio-recordings will be double coded independently (10% of recordings across both arms). Descriptive statistics will be used to report intervention fidelity and explore differences appropriately.

***SCP self-report data***

Closed questions within self-report data will be analysed using Microsoft Excel or SPSS as appropriate. Where possible descriptive statistics will be used to explore fidelity and dose. Open-ended questions will be thematically analysed using NVivo.

***Non-participant questionnaires***

Questionnaires completed by those who decline with contain open-ended questions. Open-ended questions will be thematically analysed using NVivo.

**Sample size and justification**

Our sample size is predetermined as it is building on an existing lung health study so our power calculation focusses on the size of effect that we will be able to detect given this sample size. This has been determined as follows:

- The available sample size will be smokers who attend for screening: based on telephone market surveillance data conducted in Yorkshire in May 2016, it is estimated that approximately 1083 people attending for screening over the two-year period will be eligible smokers. After excluding approximately 43 who attend during the initial 3-month trial development period (we have stated that we will allow time for the intervention to be refined, if necessary, at the start of the trial), this leaves 1040 entering the definitive trial. The sample size for our study will therefore be 1040 individuals, equally pre-randomised to two groups each of size 520
- Analysis will be by intention to treat – that is, all randomised participants will be included in the analysis regardless of whether follow-up smoking data was collected on them. Those who do not have data on smoking at follow-up will be assumed to be continuing to smoke. This is standard practice in smoking cessation research. Hence our final sample size will be 1040.
- To determine the size of effect that we can detect, we next need an estimate of the cessation rate in the usual care group. Those in the usual care group will be provided with a service similar to that provided by national smoking cessation services (NHS SSS). A 2010 systematic review of relapse rates among smokers quitting with national NHS SSS found that 53% had quit at one month, and 15% at one year. Fitting an exponential decay function to these figures we estimate a monthly relapse rate, after one month, of 11.5%. Applying these figures to our study design we would expect a quit rate of 41% at three months in those receiving standard best care.
- If the cessation rate is 41% in the usual care group, a study of 1040 individuals will provide 90% power to detect an increase to 51% (ie an increase of 10%), and 80% power to detect an increase to 49.6% (ie an increase of 8.6%). It will be able to detect smaller increases with lower power. Since 80% is generally considered the minimum acceptable power, the study will be able to detect an increase of 8.6%. This is similar to the size of effect found in the Parkes study which trialled an intervention closest in design to our intended intervention, suggesting that our trial is of adequate size to detect a realistic effect size.
- In fact, the difference we will be able to detect depends on the actual cessation rate in the usual care group. The actual cessation rate in the usual care group is likely to be lower than 41% because we are randomising all those who attend for lung health screening including some who refuse smoking cessation support (because we are interested in the effect of introducing smoking cessation support into lung cancer screening). All of these will be included in our intention to treat analysis and will be presumed to be smokers if they do not provide follow-up smoking cessation data. If the cessation rate in the usual care group is lower than 41%, we will be able to detect differences smaller than 8.6%. For example, if we assume that 30% refuse cessation support (as in similar trials) and these don’t provide follow-up data and are assumed to be smokers, the cessation rate at 3 months for usual care would be estimated at around 12% in which case we would have 80% power to detect an increase to 18.3% (6.3 % difference) in the intervention group. This shows us that it is likely that we will be able to detect effects even smaller than 8.6%.

**Assessment of efficacy**

**Primary outcome**

The primary outcome of this study will be 7-day point prevalent CO validated smoking cessation three months after the Lung Health Check

**Secondary outcomes**

•	Self-reported continuous smoking cessation at three months

•	Self-reported continuous cessation at 12 months

•	CO validated cessation at 12 months

•	Self-reported continuous cessation at four weeks

•	CO validated cessation at four weeks

We will estimate the effect of the intervention by comparing our primary and secondary outcomes between intervention and standard best practice groups using multiple logistic regression, presenting results in terms of the % achieving abstinence in the two groups, the risk difference, and the odds ratio, with 95% confidence intervals.

Primary analysis will assume that missing data implies smoking, but we will conduct sensitivity analysis to examine the impact on our findings of assuming alternative assumptions, including the Hedeker approach which assumes alternative relationships between having missing data and smoking, and using multiple imputation to test the alternative assumption that the data are missing at random (i.e. missing data can be modelled using measured variables).

•	Psychological variables, including perceived risk of lung cancer, motivation to quit smoking tobacco, confidence and efficacy beliefs (self and response).

In secondary analysis, we will also assess the effect of adjusting for potentially important

prognostic variables including lung cancer diagnosis and psychological variables as outlined

above.

In addition to intention to treat analysis, we will conduct per protocol analysis including only those who consent to take part at 4 weeks; this will enable us to compare the effect of the enhanced intervention with usual care in those who agreed to receive it. We will compare cessation rates at 4 weeks between the intervention and standard best practice groups with those not eligible for the Lung Health Check to delineate the effect of both smoking cessation interventions within the YLST.

**Assessment of safety**

Since the main trial intervention is non-pharmacological and non-invasive, no significant safety issues are anticipated. Adverse events arising from use of NRT or other licensed cessation pharmacotherapy will be managed in accordance with standard clinical practice. Electronic cigarette use by participants will be recorded and participants will be able to report their experience at the 4 week, 3 month and 12 month follow up assessments.

**Procedures for missing, unused and spurious data**

Primary analysis will be by intention-to-treat, including all those pre-randomised at Lung Health Check to usual care or intervention, and assuming, as usual in smoking cessation trials, that those who do not provide data on smoking status at 4 weeks, 3 months or 12 months, have continued to smoke. We will compare descriptively the baseline characteristics of the two treatment groups. We will estimate the proportion consenting to take part in the study (with a 95% confidence interval) at four weeks, and compare these proportions between treatment groups and according to baseline characteristics. For those who consent, we will estimate the proportion who are retained in the study and provide data up to three, 12 and 24 months and compare the characteristics of those who provide outcome data and those who do not. We will estimate the effect of the intervention by comparing our primary and secondary outcomes between intervention and standard best practice groups using multiple logistic regression, presenting results in terms of the % achieving abstinence in the two groups, the risk difference, and the odds ratio, with 95% confidence intervals. Primary analysis will assume that missing data implies smoking, but we will conduct sensitivity analysis to examine the impact on our findings of assuming alternative assumptions, including the Hedeker approach which assumes alternative relationships between having missing data and smoking, and using multiple imputation to test the alternative assumption that the data are missing at random (i.e. missing data can be modelled using measured variables). In secondary analysis, we will also assess the effect of adjusting for potentially important prognostic variables including lung cancer diagnosis and psychological variables as outlined in the secondary outcomes above. In addition to intention to treat analysis, we will conduct per protocol analysis including only those who consent to take part at 4 weeks; this will enable us to compare the effect of the enhanced intervention with usual care in those who agreed to receive it. We will compare cessation rates at 4 weeks between the intervention and standard best practice groups with those not eligible for the Lung Health Check to delineate the effect of both smoking cessation interventions within the YLST . Analysis will be conducted in Stata Version 14.

**Definition of populations analysed**

All analyses will be carried out on an intention to treat basis, including all patients who are randomised. Missing data at 4 weeks and/or 3 months will be handled as described above.

**ADVERSE EVENTS**

The occurrence of an adverse event as a result of participation within this study is not expected and as such no adverse event data will be collected.

**ETHICAL AND REGULATORY ASPECTS**

**ETHICS COMMITTEE AND REGULATORY APPROVALS**

The trial will not be initiated before the protocol, informed consent forms and participant and GP information sheets have received approval / favourable opinion from the Research Ethics Committee (REC), the respective National Health Service (NHS) or other healthcare provider’s Research & Development (R&D) department, and the Health Research Authority (HRA) if required. Should a protocol amendment be made that requires REC approval, the changes in the protocol will not be instituted until the amendment and revised informed consent forms and participant information sheets (if appropriate) have been reviewed and received approval / favourable opinion from the REC and R&D departments. A protocol amendment intended to eliminate an apparent immediate hazard to participants may be implemented immediately providing that the REC are notified as soon as possible and an approval is requested. Minor protocol amendments only for logistical or administrative changes may be implemented immediately; and the REC will be informed.

The trial will be conducted in accordance with the ethical principles that have their origin in the Declaration of Helsinki, 1996; the principles of Good Clinical Practice, and the Department of Health Research Governance Framework for Health and Social care, 2005.

**INFORMED CONSENT AND PARTICIPANT INFORMATION**

The process for obtaining participant informed consent will be in accordance with the REC guidance, and Good Clinical Practice (GCP) and any other regulatory requirements that might be introduced. The investigator or their nominee and the participant shall both sign and date the Informed Consent Form before the person can participate in the study.

The participant will receive a copy of the signed and dated forms and the original will be retained in the Trial Master File.

The decision regarding participation in the study is entirely voluntary. The investigator or their nominee shall emphasize to them that consent regarding study participation may be withdrawn at any time without penalty or affecting the quality or quantity of their future medical care, or loss of benefits to which the participant is otherwise entitled. No trial-specific interventions will be done before informed consent has been obtained.

The investigator will inform the participant of any relevant information that becomes available during the course of the study, and will discuss with them, whether they wish to continue with the study. If applicable they will be asked to sign revised consent forms.

If the Informed Consent Form is amended during the study, the investigator shall follow all applicable regulatory requirements pertaining to approval of the amended Informed Consent Form by the REC and use of the amended form (including for ongoing participants).

**RECORDS**

**Case Report Forms**

Each participant will be assigned a trial identity code number for use on CRFs other trial documents and the electronic database. The documents and database will only use their initials and study code number). Patient information will directly entered onto an eForm as part of the YLST, this information will be made accessible to the YESS Research Team under the remit of the University of Nottingham’s Handling Restricted Data Policy due to be implemented at the end of May 2018.

CRFs and eForms will be treated as confidential documents and held securely in accordance with regulations. The investigator will make a separate confidential record of the participant’s name, date of birth, local hospital number or NHS number, and Participant Trial Number (the Trial Recruitment Log), to permit identification of all participants enrolled in the trial, in accordance with regulatory requirements and for follow-up as required

CRFs shall be restricted to those personnel approved by the Chief or local Principal Investigator and recorded on the ‘Trial Delegation Log.’

All paper forms shall be filled in using black ballpoint pen. Errors shall be lined out but not obliterated by using correction fluid and the correction inserted, initialled and dated.

The Chief or local Principal Investigator shall sign a declaration ensuring accuracy of data recorded in the CRF.

**Source documents**

Source documents shall be filed at the investigator’s site and may include but are not limited to, consent forms, current medical records, laboratory results and records. A CRF may also completely serve as its own source data. Only trial staff as listed on the Delegation Log shall have access to trial documentation other than the regulatory requirements listed below.

**Direct access to source data / documents**

The CRF and all source documents, including progress notes and copies of laboratory and medical test results shall made be available at all times for review by the Chief Investigator, Sponsor’s designee and inspection by relevant regulatory authorities (e.g. DH, Human Tissue Authority).

**DATA PROTECTION**

All trial staff and investigators will endeavour to protect the rights of the trial’s participants to privacy and informed consent, and will adhere to the Data Protection Act, 1998. The CRF will only collect the minimum required information for the purposes of the trial. CRFs will be held securely, in a locked room, or locked cupboard or cabinet. Access to the information will be limited to the trial staff and investigators and relevant regulatory authorities (see above). Computer held data including the trial database will be held securely and password protected. All data will be stored on a secure dedicated web server. Access will be restricted by user identifiers and passwords (encrypted using a one way encryption method).

Information about the trial in the participant’s medical records / hospital notes will be treated confidentially in the same way as all other confidential medical information.

Electronic data will be backed up every 24 hours to both local and remote media in encrypted format.

**QUALITY ASSURANCE & AUDIT**

**INSURANCE AND INDEMNITY**

Insurance and indemnity for trial participants and trial staff is covered within the NHS Indemnity Arrangements for clinical negligence claims in the NHS, issued under cover of HSG (96)48. There are no special compensation arrangements, but trial participants may have recourse through the NHS complaints procedures.

The University of Nottingham as research Sponsor indemnifies its staff, research participants and research protocols with both public liability insurance and clinical trials insurance. These policies include provision for indemnity in the event of a successful litigious claim for proven non-negligent harm.

**TRIAL CONDUCT**

Trial conduct may be subject to systems audit of the Trial Master File for inclusion of essential documents; permissions to conduct the trial; Trial Delegation Log; CVs of trial staff and training received; local document control procedures; consent procedures and recruitment logs; adherence to procedures defined in the protocol (e.g. inclusion / exclusion criteria, correct randomisation, timeliness of visits); adverse event recording and reporting; accountability of trial materials and equipment calibration logs.

**TRIAL DATA**

Monitoring of trial data shall include confirmation of informed consent; source data verification; data storage and data transfer procedures; local quality control checks and procedures, back-up and disaster recovery of any local databases and validation of data manipulation.

Entries on CRFs will be verified by inspection against the source data. A sample of CRFs (10% or as per the study risk assessment) will be checked on a regular basis for verification of all entries made. In addition the subsequent capture of the data on the trial database will be checked. Where corrections are required these will carry a full audit trail and justification.

Trial data and evidence of monitoring and systems audits will be made available for inspection by REC as required. Interview transcripts will be checked against audio-recordings for accuracy. Trial data will be stored and maintained on Nottingham or Cardiff servers. When and if data is transferred it will be conducted in a controlled/verified manner in accordance with a data management plan and the University of Nottingham’s ‘Handling Personal Data’ policy

**RECORD RETENTION AND ARCHIVING**

In compliance with the ICH/GCP guidelines, regulations and in accordance with the University of Nottingham Research Code of Conduct and Research Ethics, the Chief or local Principal Investigator will maintain all records and documents regarding the conduct of the study. These will be retained for at least 7 years or for longer if required. If the responsible investigator is no longer able to maintain the study records, a second person will be nominated to take over this responsibility.

The Trial Master File and trial documents held by the Chief Investigator on behalf of the Sponsor shall be finally archived at secure archive facilities at the University of Nottingham. This archive shall include all trial databases and associated meta-data encryption codes.

**DISCONTINUATION OF THE TRIAL BY THE SPONSOR**

The Sponsor reserves the right to discontinue this trial at any time for failure to meet expected enrolment goals, for safety or any other administrative reasons. The Sponsor shall take advice from the Trial Steering Committee and Data Monitoring Committee as appropriate in making this decision.

**STATEMENT OF CONFIDENTIALITY**

Individual participant medical information obtained as a result of this study are considered confidential and disclosure to third parties is prohibited with the exceptions noted above.

Participant confidentiality will be further ensured by utilising identification code numbers to correspond to treatment data in the computer files.

Such medical information may be given to the participant’s medical team and all appropriate medical personnel responsible for the participant’s welfare.

If information is disclosed during the study that could pose a risk of harm to the participant or others, the researcher will discuss this with the CI and where appropriate report accordingly.

Data generated as a result of this trial will be available for inspection on request by the participating physicians, the University of Nottingham representatives, the REC, local R&D Departments and the regulatory authorities.

**PUBLICATION AND DISSEMINATION POLICY**

The results of the study will be presented at appropriate conferences and submitted for publication in a peer-reviewed journal.

**USER AND PUBLIC INVOLVEMENT**

A Yorkshire lay representative previously diagnosed with lung cancer has contributed to the design of the study and we will engage with the patient/public representative group established as part of the YLST to contribute to the direction and governance of the project. We have conducted pre-development work with smokers or ex-smokers and individuals who have experience of lung and other cancers from Nottingham, South Wales and Bradford, and will seek to test the developed intervention with smokers or ex-smokers and individuals who have experience of lung and other cancers recruited from the Yorkshire area before the trial commences. Representatives will be reimbursed for their time spent on study activities and training will be provide for those who have not previously participated in research.

**STUDY FINANCES**

**Funding source**

This study is funded by Yorkshire Cancer Research (NOT414)

**Participant stipends and payments**

Participants will not be paid to participate in the trial and no expenses will be incurred as a consequence of participation. Participants in the process evaluation will be provided with a shopping voucher worth £20 for longer face-to face interviews, and £10 for interviews conducted over the telephone as recompense for the inconvenience of participation.

**REFERENCES**

1.	Public Health England. Healthier Lives, Premature Mortality [Available from: http://healthierlives.phe.org.uk/topic/mortality.

2.	Riaz SP, Horton M, Kang J, Mak V, Luchtenborg M, Moller H. Lung cancer incidence and survival in England: an analysis by socioeconomic deprivation and urbanization. J Thorac Oncol. 2011;6(12):2005-10.

3.	Office for National Statistics. Chapter 1 - Smoking (General Lifestyle Survey Overview - a report on the 2011 General Lifestyle Survey) 2013 [Available from: http://www.ons.gov.uk/ons/dcp171776_302558.pdf.

4.	NHS England and Public Health England. The Cancer Dashoard [Available from: https://www.cancerdata.nhs.uk/dashboard/lung.html#?tab=Overview&ccg=03G.

5.	Humphrey LL, Teutsch S, Johnson M. Lung cancer screening with sputum cytologic examination, chest radiography, and computed tomography: an update for the US Preventive Services Task Force. Annals of Internal Medicine. 2004;140(9):740-53.

6.	van Klaveren RJ, Oudkerk M, Prokop M, Scholten ET, Nackaerts K, Vernhout R, et al. Management of lung nodules detected by volume CT scanning. N Engl J Med. 2009;361(23):2221-9.

7.	Field JK, Devaraj A, Duffy SW, Baldwin DR. CT screening for lung cancer: Is the evidence strong enough? Lung Cancer. 2016;91:29-35.

8.	Field JK, Smith RA, Aberle DR, Oudkerk M, Baldwin DR, Yankelevitz D, et al. International association for the study of lung cancer computed tomography screening workshop 2011 report. Journal of Thoracic Oncology. 2012;7(1):10-9.

9.	NHS Choices. Lung cancer-Causes 2015 [Available from: http://www.nhs.uk/Conditions/Cancer-of-the-lung/Pages/Causes.aspx.

10.	Doll R, Peto R, Boreham J, Sutherland I. Mortality in relation to smoking: 50 years' observations on male British doctors. Bmj. 2004;328(7455):1519.

11.	Halpern MT, Gillespie BW, Warner KE. Patterns of absolute risk of lung cancer mortality in former smokers. J Natl Cancer Inst. 1993;85(6):457-64.

12.	Pedersen JH, Tonnesen P, Ashraf H. Smoking cessation and lung cancer screening. Ann Transl Med. 2016;4(8):157.

13.	Zeliadt SB, Heffner JL, Sayre G, Klein DE, Simons C, Williams J, et al. Attitudes and Perceptions About Smoking Cessation in the Context of Lung Cancer Screening. JAMA Intern Med. 2015;175(9):1530-7.

14.	McBride CM, Emmons KM, Lipkus IM. Understanding the potential of teachable moments: the case of smoking cessation. Health Educ Res. 2003;18(2):156-70.

15.	Pineiro B, Simmons VN, Palmer AM, Correa JB, Brandon TH. Smoking cessation interventions within the context of Low-Dose Computed Tomography lung cancer screening: A systematic review. Lung Cancer. 2016;98:91-8.

16.	Quaife SL, Marlow LA, McEwen A, Janes SM, Wardle J. Attitudes towards lung cancer screening in socioeconomically deprived and heavy smoking communities: informing screening communication. Health Expect. 2016.

17.	van der Aalst CM, van den Bergh KAM, Willemsen MC, de Koning HJ, van Klaveren RJ. Lung cancer screening and smoking abstinence: 2 year follow-up data from the Dutch–Belgian randomised controlled lung cancer screening trial. Thorax. 2010;65(7):600-5.

18.	Ashraf H, Saghir Z, Dirksen A, Pedersen JH, Thomsen LH, Dossing M, et al. Smoking habits in the randomised Danish Lung Cancer Screening Trial with low-dose CT: final results after a 5-year screening programme. Thorax. 2014;69(6):574-9.

19.	Brain K, Carter B, Lifford KJ, O. B, Devaraj A, Baldwin D, et al. Impact of low-dose CT screening on smoking cessation among high-risk participants in the UK Lung Cancer Screening trial. Thorax. 2017 In Press.

20.	Tanner NT, Kanodra NM, Gebregziabher M, Payne E, Halbert CH, Warren GW, et al. The Association between Smoking Abstinence and Mortality in the National Lung Screening Trial. Am J Respir Crit Care Med. 2016;193(5):534-41.

21.	Tammemägi MC, Berg CD, Riley TL, Cunningham CR, Taylor KL. Impact of lung cancer screening results on smoking cessation. Journal of the National Cancer Institute. 2014;106(6):dju084.

22.	Park ER, Gareen IF, Japuntich S, Lennes I, Hyland K, DeMello S, et al. Primary Care Provider-Delivered Smoking Cessation Interventions and Smoking Cessation Among Participants in the National Lung Screening Trial. JAMA Intern Med. 2015;175(9):1509-16.

23.	Villanti AC, Jiang Y, Abrams DB, Pyenson BS. A cost-utility analysis of lung cancer screening and the additional benefits of incorporating smoking cessation interventions. PLoS One. 2013;8(8):e71379.

24.	Goffin JR, Flanagan WM, Miller AB, Fitzgerald NR, Memon S, Wolfson MC, et al. Cost-effectiveness of Lung Cancer Screening in Canada. JAMA Oncol. 2015;1(6):807-13.

25.	Hartmann-Boyce J, Lancaster T, Stead LF. Print-based self-help interventions for smoking cessation. The Cochrane database of systematic reviews. 2014(6):CD001118.

26.	Marshall HM, Courtney DA, Passmore LH, McCaul EM, Yang IA, Bowman RV, et al. Brief Tailored Smoking Cessation Counseling in a Lung Cancer Screening Population is Feasible: A Pilot Randomized Controlled Trial. Nicotine Tob Res. 2016;18(7):1665-9.

27.	Murray R, Leonardi-Bee J, Marsh J, Jayes L, Li J, Parrott S, et al. Systematic identification and treatment of smokers by hospital based cessation practitioners in a secondary care setting: cluster randomised controlled trial. BMJ. 2013;347:f4004.

28.	Campbell KA, Cooper S, Fahy SJ, Bowker K, Leonardi-Bee J, McEwen A, et al. 'Opt-out' referrals after identifying pregnant smokers using exhaled air carbon monoxide: impact on engagement with smoking cessation support. Tob Control. 2017;26(3):300-6.

29.	Nahhas GJ, Cummings KM, Talbot V, Carpenter MJ, Toll BA, Warren GW. Who Opted Out of an Opt-Out Smoking-Cessation Programme for Hospitalised Patients? Journal of Smoking Cessation. 2016.

30.	National Institute for Health and Care Excellence. Smoking: stopping in pregnancy and after childbirth 2010 [Available from: <https://www.nice.org.uk/guidance/PH26/chapter/1-Recommendations#recommendation-1-identifying-pregnant-women-who-smoke-and-referring-them-to-nhs-stop-smoking>.

31.	National Institute for Health and Care Excellence. Smoking: acute, maternity and mental health services 2013 [7th March 2017]. Available from: <https://www.nice.org.uk/guidance/ph48/resources/smoking-acute-maternity-and-mental-health-services-1996364658373>.

32.	Gilbert H, Sutton S, Morris R, Petersen I, Galton S, Wu Q, et al. Effectiveness of personalised risk information and taster sessions to increase the uptake of smoking cessation services (Start2quit): a randomised controlled trial. Lancet. 2017;389(10071):823-33.

33.	Farsalinos KE, Spyrou A, Tsimopoulou K, Stefopoulos C, Romagna G, Voudris V. Nicotine absorption from electronic cigarette use: comparison between first and new-generation devices. Scientific Reports. 2014;4:4133.

34.	National Centre for Smoking Cessation and Training. E-cigaratte briefing. http://www.ncsct.co.uk/usr/pub/e-cigarette_briefing.pdf; 2014.

35.	DIRECTIVE 2014/40/EU OF THE EUROPEAN PARLIAMENT AND OF THE COUNCIL on the approximation of the laws, regulations and administrative provisions of the Member States concerning the manufacture, presentation and sale of tobacco and related products and repealing Directive 2001., (3 April 2014).

36.	Medicines & Healthcare products Regulatory Agency (MRHA). E-cigarettes: regulations for consumer products, Guidance on how to get an e-cigarette on the market in the UK, including the notification scheme and reporting problems with e-cigarettes. In: Medicines & Healthcare products Regulatory Agency, editor. London2016.

37.	NCSCT. Certfied Practioner Training,. <http://elearningncsctcouk/england>. Accessed 2016.

38.	Medicines Complete BNF. Nicotine Indications and Dose [updated 2016. Available from: <https://www.medicinescomplete.com/mc/bnf/current/PHP3201-nicotine.htm#PHP46731-side-effects>.

39.	Royal College of Physicians. Nicotine without smoke: Tobacco harm reduction. London; 2016.

**3. Supplementary Document 3: Yorkshire Enhanced Stop Smoking Study Amendment Log**

| **Amendment Number** | **Detail of Amendment** | **Protocol Number** | **Category** | **Date of submission** | **Approval Date** |
| --- | --- | --- | --- | --- | --- |
| 1. | Re-format consent forms so that they fit onto one piece of paper to make printing easier. | V2 18 07 2018 | Minor | 16/10/2018 | 16/10/2018 |
| 2. | **Letters to GP**  We realised that our GP letter for contraindication for NRT was not fit for purpose. We proposed an amendment to the letter to only ask the GP if they feel that the patient may be contraindicated for NRT and have created three new separate documents to request pharmacotherapies. Two documents are letters requesting stage one and stage two Varenicline - these letters align closely with the current practice of the local stop smoking service in Leeds. The third document is a health assessment questionnaire to be completed by SCPs with the patient to check for contraindications for pharmacotherapies. These will then be included with the letter to request pharmacotherapies from the GP.  **Minor amendments to interview topic guides**  To include questions around participation in the lung health check and influences on smoking, and opinions on the approach from SCPs on the mobile unit.  **Make incentives clearer for inconvenience of taking part in the interviews.**  We have added to the protocol that we will provide a £20 shopping voucher for longer face-to-face interviews, and a £10 shopping voucher if interviews are completed over the telephone.  **Audio recording of initial consultation with patients on the screening van.**  We propose to record one consultation per SCP per week on the mobile unit to check the fidelity of the support that we offer to patients. | V2.1 04 02 2019 | Substantial | 01/03/2019 | 29/03/2019 |
| 3. | Submit YESS Sub-section 1 & 2 Participant Information Sheet v1.0 17 05 2019 as it had been omitted from the original application. | V2.3 04/02/2019 | Substantial | 05/06/2019 | 26/07/2019 |
| 4. | Reduction of recruitment target from 2019 to 1040 to realistically reflect the population. We have accordingly revised our power calculations and after excluding approximately 43 who attend during the initial 3-month trial development period, this leaves 1040 entering the definitive trial. With intention-to-treat analysis of all 1040 (520 per group) the study will have 90% power to detect a 10 percentage point higher quit rate at three months. | V2.4 04 09 2019 | Substantial | 04/11/2019 | 11/11/2019 |
| 5. | Recruitment end date changed to 07/11/2020 | V2.4 04 09 2019 | Non Substantial | 09/01/2020 | 09/01/2020 |
| 6. | Changes to consent and intervention delivery to include ‘over telephone’ if patients are self-isolating to protect against COVID-19. | V2.4 16/03/2020 | Non substantial | 16/03/2020 | 16/03/2020 |
| 7. | Recruitment extended from August 31^st^ 2020 to February 2021 and data collection to 14^th^ March 2022 to accommodate months lost to COVID-19, | V2.4 16/03/2020 | Non Substantial | 17/06/2020 | 18/06/2020 |
| 8. | Inclusion of outcome; 7 day point prevalent self-reported quit at three months and amended process evaluation topic guides to include reactions to COVID | V2.4 16/07/2020 | Non substantial | 17/07/2020 | 21/07/2020 |

**4. Supplementary Table 1:** Baseline characteristics of intervention and standard best practice study arms according to completion of follow-up data collection measures.

|  | Intervention ^1^LTF  (n=182) | Intervention completed  (n=345) | ^2^SBP  LTF  (n=175) | SBP completed  (n=301) |
| --- | --- | --- | --- | --- |
| Age (yrs.) |  |  |  |  |
| Mean ^3^(SD) | 63.9 (6.7) | 65.7 (6.4) | 64.3 (6.7) | 65.8 (6.7) |
| Median ^4^(IQR) | 63 (58-67) | 65 (61-70) | 63 (59-68.5) | 66 (60-71) |
| Sex |  |  |  |  |
| Male | 77 (42) | 174 (50) | 107 (61) | 157 (52) |
| Female | 105 (58) | 171 (50) | 68 (39) | 144 (48) |
| Ethnicity |  |  |  |  |
| White | 170 (93) | 332 (96) | 169 (97) | 387 (96) |
| Black | 4 (2.2) | 3 (0.9) | 2 (1.1) | 5 (1.7) |
| Asian | 5 (2.7) | 6 (1.7) | 2 (1.1) | 4 (1.3) |
| Others | 3 (1.6) | 4 (1.2) | 1 (0.6) | 3 (1) |
| Unknown | 0 | 0 | 1 (0.6) | 2 (0.7) |
| ^5^IMD score, quintiles |  |  |  |  |
| 1 (most deprived) | 81 (44) | 110 (31.5) | 87 (51) | 116 (38.3) |
| 2 | 28 (15.4) | 54 (15.7) | 39 (22.1) | 49 (17.6) |
| 3 | 36 (19.3) | 70 (20) | 26 (14.8) | 43 (14.2) |
| 4 | 29 (15.9) | 66 (19.1) | 10 (5.7) | 67 (22.3) |
| 5 (least deprived) | 8 (4.3) | 45 (13) | 11 (6.3) | 26 (8.7) |
| Level of education |  |  |  |  |
| No qualifications | 102 (56) | 180 (52) | 109 (62) | 144 (48) |
| O-levels, ^6^CSE, or ^7^GCSE | 51 (28) | 79 (23) | 33 (19) | 70 (23) |
| A-level and above | 28 (15.3) | 85 (24.7) | 32 (18.3) | 83 (28) |
| Unknown | 1 (0.5) | 1 (0.3) | 1 (0.6) | 3 (1) |
| Family history of lung cancer | 38 (21) | 59 (17) | 39 (22) | 57 (19) |
| ^8^CO reading (^9^ppm) |  |  |  |  |
| Mean (SD) | 16.8 (7.9) | 16 (9.6) | 17.2 (10.5) | 16 (10.2) |
| Median (IQR) | 16 (11-22) | 15 (10-21) | 16 (11-22) | 14 (9-20) |
| Unknown | 0 | 0 | 2 (1.2) | 0 |
| Age started smoking (yrs.) |  |  |  |  |
| Mean (SD) | 16.2 (4.4) | 16.2 (4.3) | 16.4 (4) | 16.2 (3.7) |
| Median (IQR) | 15 (14-18) | 16 (14-18) | 16 (14-18) | 16 (14-18) |
| Unknown | 0 | 0 | 2 (1.2) | 0 |
| Fagerstrom test of nicotine dependency | |  |  |  |
| <4 (less dependent) | 51 (28) | 127 (36.8) | 52 (29.7) | 115 (38.2) |
| 4-6 (moderately dependent) | 80 (44) | 152 (44.1) | 94 (53.7) | 123 (40.8) |
| 7-10 (highly dependent) | 34 (18.7) | 40 (11.6) | 19 (10.9) | 39 (13) |
| Unknown | 17 (9.3) | 26 (7.5) | 10 (5.7) | 24 (8) |
| Cigarettes per day |  |  |  |  |
| ≤10 | 46 (8.8) | 117 (33.9) | 60 (34.3) | 115 (38.2) |
| 11-20 | 103 (56.6) | 168 (48.7) | 83 (47.4) | 130 (43.2) |
| 21-30 | 20 (11) | 38 (11) | 20 (11.4) | 36 (12) |
| >30 | 5 (2.7) | 6 (1.7) | 4 (2.3) | 8 (2.7) |
| Unknown | 8 (4.4) | 16 (4.6) | 8 (4.6) | 12 (4) |
| Scan outcome |  |  |  |  |
| Negative | 150 (82.4) | 274 (79.4) | 141 (80.6) | 231 (76.7) |
| Indeterminate | 24 (13.2) | 62 (18) | 28 (16) | 65 (21.6) |
| Positive | 8 (4.3) | 9 (2.6) | 6 (3.6) | 5 (1.7) |
| Motivation to quit smoking | | | | |
| No intention/Intention to quit but don't want to | 64 (35.6) | 102 (30) | 54 (31.2) | 101 (33.6) |
| Intention to quit but haven't thought when | 60 (33) | 101 (29.3) | 58 (34) | 112 (37.2) |
| Intention to quit and hope soon | 26 (14) | 57 (17) | 26 (15) | 45 (15) |
| Intention to quit in the next 1 to 3 months | 31 (17.5) | 62 (17.9) | 33 (18.6) | 40 (13.2) |
| Already quit | 0 | 6 (1.7) | 2 (1.2) | 3 (1) |
| Unknown | 1 (0.5) | 7 (2) | 2 (1.2) | 0 |
| Times tried to quit the past year |  |  |  |  |
| Mean (SD) | 0.6 (1.9) | 0.6 (1.3) | 0.6 (1.1) | 0.8 (3.1) |
| Median (IQR) | 0 (0-1) | 0 (0-1) | 0 (0-1) | 0 (0-1) |
| Unknown | 1 (0.5) | 2 (0.7) | 2 (1.2) | 0 |
| Self-efficacy of quitting smoking | | | | |
| How confident are you that you can quit? |  |  |  |  |
| Extremely/Moderately | 92 (50) | 175 (51) | 80 (46) | 153 (51) |
| Somewhat/Slightly | 56 (31) | 122 (37) | 58 (33) | 106 (36) |
| Not at all | 31 (17) | 42 (12) | 34 (20) | 44 (15) |
| Unknown | 3 (1.6) | 6 (1.8) | 3 (1.8) | 3 (1) |
| 7-day point prevalent abstinent | | | | |
| 4-weeks, (validated) | 39 (21.4) | 98 (28.4) | 36 (20.6) | 87 (28.9) |
| 4-weeks, (self-reported) | 47 (25.8) | 108 (31.3) | 44 (25.1) | 92 (30.6) |

*¹ Lost to Follow Up*

*^2^Standard Best Practice*

*^3^Standard Deviation*

*^4^Inter Quartile Range,*

*^5^Index of Multiple Deprivation*

*^6^Certificate of Secondary Education (1965 -1987)*

*^7^General Certificate of Secondary Education (1988-present)*

*^8^Carbon Mononxide*

*^9^Parts per Million*

**5. Supplementary Table 2: Quit status in those CO-verified abstinent from smoking according at 3-months according to quit status at 4-weeks**

|  | Intervention (n=137) | ^1^SBP  (n=173) | Risk difference % (95% CI) | Unadjusted OR (95% CI) |
| --- | --- | --- | --- | --- |
| ^2^CO-verified quit at 4-weeks | 80 (58.4) | 73 (59.3) | -1.0  (-12.9 - 11.0) | 0.96  (0.59 - 1.58) |
| Not quit at 4-weeks | 97 (24.5) | 70 (19.8) | 5.1  (-0.2 – 10.9) | 1.34  (0.95 – 1.89) |

*^1^Standard Best Practice*

*^2^Carbon Monoxide*

**6. Supplementary Table 3 Subgroup analysis of validated 7-day point prevalence abstinence after 12-months**

| Outcomes | Intervention (n=527) | ^1^SBP (n=476) | Risk difference % (95%CI) | Unadjusted OR (95%CI) | p-value |
| --- | --- | --- | --- | --- | --- |
| Sex |  |  |  |  | 0.011* |
| Male | 83 (29.7) | 79 (35.1) | -5.36  (-13.6 - 2.86) | 0.78  (0.54-1.14) | 0.2 |
| Female | 71 (28.6) | 57 (22.7) | 5.91  (-1.72 - 13.6) | 1.36  (0.92-2.04) | 0.131 |
| Age (yrs.) |  |  |  |  | 0.835* |
| 55-60 | 49 (32) | 43 (29.7) | 2.37  (-8.11 - 12.85) | 1.12  (0.68-1.83) | 0.658 |
| 61-65 | 35 (25) | 28 (26.7) | -0.7  (-11.7 - 10.3) | 0.91  (0.51-1.64) | 0.768 |
| 66-70 | 37 (33.3) | 35 (32.4) | 0.9  (-11.5 - 13.4) | 1.04  (0.59-1.84) | 0.884 |
| 71-83 | 33 (26.8) | 30 (25.4) | 1.4  (-9.68 - 12.5) | 1.07  (0.61-1.92) | 0.804 |
| ^2^IMD |  |  |  |  | 0.109* |
| Most deprived | 51 (26.7) | 51 (24.9) | 1.82  (-6.8 - 10.4) | 1.10  (0.70-1.72) | 0.678 |
| - | 20 (24.4) | 25 (28.4) | -4  (-17.2 - 9.21) | 0.81  (0.40-1.61) | 0.553 |
| - | 30 (28.3) | 19 (27.5) | 0.8  (-12.8 - 14.3) | 1.04  (0.53-2.06) | 0.912 |
| - | 29 (30.5) | 30 (39) | -8.43  (-22.7 - 5.86) | 0.69  (0.36-1.29) | 0.247 |
| Least deprived | 24 (45.3) | 11 (29.7) | 11.2  (-9.4 – 31.8) | 1.95  (0.82-4.87) | 0.293 |
| Level of education |  |  |  |  | 0.161* |
| None | 71 (27.2) | 68 (26.9) | 1.7  (-5.75 - 9.15) | 0.99  (0.72-1.36) | 0.947 |
| O-levels, CSE, or GCSE | 45 (34.6) | 33 (32) | 2.57  (-9.6 - 14.7) | 1.12  (0.65-1.95) | 0.679 |
| Above | 37 (32.7) | 34 (29.3) | 3.43  (-8.54 - 15.4) | 1.17  (0.67-2.06) | 0.575 |
| Scan outcome |  |  |  |  | 0.371* |
| Negative | 118 (27.8) | 106 (28.5) | -0.6  (-6.92 - 5.59) | 0.97  (0.71-1.32) | 0.835 |
| Indeterminate/Positive | 36 (35) | 30 (28.8) | 6.11  (-6.56 - 18.8) | 1.32  (0.74-2.39) | 0.347 |
| Pharmacotherapies and E-cigarettes | | |  |  | 0.132* |
| NRT | 25 (17.6) | 28 (25) | -7.39  (-17.6 - 2.78) | 0.64  (0.35-1.18) | 0.152 |
| E-cigarette | 30 (33) | 32 (33.7) | -0.70  (-14.2 - 12.8) | 0.97  (0.52-1.78) | 0.917 |
| NRT+ E-cigarette | 60 (34.3) | 52 (31.1) | 3.14  (-6.8 - 13.1) | 1.15  (0.73-1.82) | 0.535 |
| Pharmacotherapy | 16 (34.8) | 9 (24.3) | 10.4  (-9 - 30) | 1.65  (0.64-4.49) | 0.304 |
| None | 22 (31.4) | 15 (25.4) | 6  (-9.54 - 21.5) | 1.34  (0.62-2.95) | 0.453 |

**P-value for interaction*

*^1^Standard Best Practice*

*^2^Index of Multiple Deprivation*

**7. Supplementary Table 4*:* Primary and secondary outcomes by omitting from analysis those consented prior to the change in the timing of consent.**

| **Outcomes** | **Intervention**  **n=404** | **^2^SBP**  **n=362** | **Risk difference % (95%CI)** | **Unadjusted OR (95%CI)** | **p-value** |
| --- | --- | --- | --- | --- | --- |
| **7-day point prevalence abstinence** | | | | | |
| 3-months, validated | 147 (36·4) | 118 (32·6) | 3·72  (-2·33 - 9·77) | 1·18  (0·88 - 1·60) | 0·271 |
| 3-months, self-reported | 169 (41·8) | 148 (40·9) | 0·94  (-6·03 - 7·93) | 1·04  (0·78 - 1·39) | 0·790 |
| 12- months, validated | 125 (30·9) | 110 (30·4) | 0·55  (-5·98 - 7·09) | 1·03  (0·76 - 1·40) | 0·868 |
| 12- months, self-reported | 134 (33·2) | 118 (32·6) | 0·57  (-6·09 - 7·23) | 1·03  (0·76 - 1·39) | 0·866 |
| **Prolonged smoking cessation** | | | | | |
| 3-months, validated | 119 (29·5) | 93 (25·7) | 3·76  (-2·56 - 10·1) | 1·21  (0·88 - 1·66) | 0·245 |
| 3-months, self-reported | 133 (32·9) | 115 (31·8) | 1·15  (-5·48 - 7·78) | 1·05  (0·78 - 1·43) | 0·734 |
| 12- months, validated | 28 (6·9) | 17 (4·7) | 2·23  (-1·06 - 5·53) | 1·51  (0·82 - 2·86) | 0·192 |
| 12- months, self-reported | 37 (9·2) | 25 (6·9) | 2·25  (-1·58 - 6·09) | 1·36  (0·80 - 2·33) | 0·255 |
| **Attempts to quit smoking** | | | | | |
| 3-months | - | - | - | 0·94^1^  (0·81 - 1·10) | 0·434 |
| 12-months | - | - | - | 1·13^1^  (0·93 - 1·37) | 0·223 |

*^1^ The measure of effect is an unadjusted incidence rate ratio (IRR)*

*^2^Standard Best Practice*

**8. Supplementary Table 5: Primary and secondary outcomes excluding participants with outcomes measured during COVID-19 lockdown period.**

| **Outcomes** | **Intervention** | **^2^SBP** | **Risk difference % (95%CI)** | **Unadjusted OR (95%CI)** | **p-value** |
| --- | --- | --- | --- | --- | --- |
| 7-day point prevalence abstinence | | | | | |
| 3-months, validated* | 133 (30·0) | 106 (26·3) | 3·71  (-2·33 - 9·77) | 1·20  (0·89 - 1·63) | 0·230 |
| 3-months, self-reported* | 165 (37·2) | 143 (35·5) | 1·76  (-4·72 - 8·24) | 1·08  (0·81 - 1·23) | 0·595 |
| 12- months, validated^ | 143 (29·1) | 126 (28·5) | 0·55  (-5·2 – 6·37) | 1·03  (0·77 - 1·36) | 0·851 |
| 12- months, self-reported^ | 154 (31·3) | 137 (31·0) | 0·3  (-5·64 – 6·25) | 1·01  (0·77.- 1·34) | 0·920 |
| **Prolonged smoking cessation** |  |  |  |  |  |
| 3-months, validated* | 110 (24·8) | 89 (22·1) | 2·75  (-2·96 - 8·45) | 1·16  (0·62 - 1·60) | 0·347 |
| 3-months, self-reported* | 130 (29·3) | 115 (28·5) | 0·81  (-5·31 - 6·92) | 1·04  (0·77 - 1·40) | 0·795 |
| 12- months, validated^ | 30 (6·1) | 18 (4·1) | 2·04  (-0·82 - 4·91) | 1·53  (0·85 - 2·83) | 0·169 |
| 12- months, self-reported^ | 41 (8·3) | 29 (6·6) | 1·77  (-1·58 - 5·13) | 1·29  (0·79 - 2·13) | 0·305 |
| **Attempts to quit smoking** |  |  |  |  |  |
| 3-months | - | - | - | 0·94^1^  (0·81 - 1·10) | 0·412 |
| 12-months | - | - | - | 1·15^1^  (0·97 - 1·37) | 0·095 |

*^1^ The measure of effect is an unadjusted incidence rate ratio (IRR).*

**n=443 Intervention, 403 SBP at 3 months*

*^n=492 intervention, 442 SBP at 12 months*

*^2^Standard Best Practice*

**9. Supplementary Figure 1: Example personalised intervention booklet detailing scan findings of coronary artery calcification and/or emphysema**
